# SSS–DDQN solution-space best-first search for robust trajectory tracking of Mecanum robots under partial observability

**DOI:** 10.3389/frobt.2026.1896260

**Published:** 2026-09-28

**Authors:** Oorappan G M, Lakshmanan S A

**Affiliations:** 1 Robotics and Automation, KGiSL Institute of Technology, Coimbatore, Tamil Nadu, India; 2 Department of Electronics and Communication Engineering, Amrita School of Engineering, Amrita Vishwa Vidyapeetham, Chennai, Tamil Nadu, India

**Keywords:** DDQN, EKF belief state, Mecanum robot, partial observability, reinforcement learning, robust control, SSS* search, trajectory tracking

## Abstract

Robust trajectory tracking of Mecanum-wheel mobile robots under partial observability is challenging due to nonlinear motion coupling, noisy measurements, sensor dropout, and external disturbances. In this study, we propose an SSS*–DDQN solution-space best-first search controller for robust trajectory tracking of a four-Mecanum-wheel mobile robot. The proposed framework integrates extended Kalman filter (EKF)-based belief-state estimation, QMDP belief sampling, DDQN value evaluation, and SSS* OPEN-list search with LIVE/SOLVED node refinement. The EKF estimates the hidden robot state under noisy observations and dropout, the DDQN provides learned long-term value evaluation, and the SSS* search refines promising corrective-action clusters using beta-bound pruning, delayed MIN-sibling refinement, bounded OPEN-list management, and secondary lookahead. The controller is evaluated using lemniscate trajectory tracking and compared with EKF-belief PER-DDQN, QMDP-PER-DDQN, Alpha–Beta Minimax, and AB–DDQN Hybrid controllers. The proposed SSS*–DDQN achieved the best overall ranking with ${\text{RMSE}}_{xy}=0.01906$ m, ${\text{MAE}}_{xy}=0.016074$ m, and a cumulative reward of 1843.6. Compared with the AB–DDQN Hybrid baseline, it reduced ${\text{RMSE}}_{xy}$ by 12.93% and ${\text{MAE}}_{xy}$ by 17.80%. Statistical validation over multiple stochastic trials further showed the lowest mean ${\text{RMSE}}_{xy}$ of 0.01847±0.00132 m and ${\text{MAE}}_{xy}$ of 0.01554±0.00128 m, with narrow 95% confidence intervals. The results confirm that the proposed SSS*–DDQN improves robust action selection and provides consistent tracking performance under partial observability.

## 1 Introduction

Mecanum-wheel mobile robots are widely used in warehouse automation, hospital logistics, service robotics, industrial inspection, and indoor navigation because they can move longitudinally, laterally, and rotationally without changing body orientation (Klančar et al., 2017; Vasudevan, 2023; Santoro, 2021; van der Spijk, 2024; Mao, 2025). However, accurate trajectory tracking remains difficult due to nonlinear dynamics, coupled longitudinal–lateral–yaw motion, wheel–ground interaction, actuator saturation, friction variation, external disturbances, and partial observability caused by noisy or missing sensor data (Pandey and Gajbhiye, 2024; Anil et al., 2025; Banik et al., 2025; Chen, 2024; Shahgholian et al., 2024). Existing finite-time, prescribed-time, observer-based, adaptive, robust, and model-predictive control methods improve convergence, stability, and disturbance rejection (Pandey and Gajbhiye, 2024; Anil et al., 2025; Banik et al., 2025; Chen, 2024; Shahgholian et al., 2024; Hendzel and Kołodziej, 2021; Lee, 2025). Nevertheless, these methods mainly rely on predefined control laws and do not explicitly perform learned value-guided online search over future corrective actions under partial observability.

Uncertainty-aware estimation is essential when the true robot state is affected by sensor noise, dropout, disturbance, or occlusion. Landmark-based tracking, fuzzy distributed extended Kalman filter (EKF), robust landmark selection, and uncertainty-aware navigation have shown the importance of state estimation and belief handling in robotic systems (Taheri and Xia, 2020; Tsai et al., 2026b; Beinhofer et al., 2013; Yao et al., 2024; Yao et al., 2025b; Yao et al., 2025a). Reinforcement learning further provides an adaptive framework for robot control and navigation by learning corrective actions from interaction data (Saidi and Telmoudi, 2026; Ben Hazem et al., 2025; López-González et al., 2025; Vazquez-Santiago, 2025). Recent RL-based navigation and planning methods, including frontier exploration, PPO-GRU-based Mecanum path planning, robust RL, fuzzy TD3 with GRU-attention, recurrent SAC, and DRL-based navigation, demonstrate the growing role of learning-based decision-making in dynamic environments (Wang et al., 2024b; An et al., 2026; Jestel et al., 2026; Tsai et al., 2026a; Yu et al., 2023; Li et al., 2019; Li et al., 2024; Zhou et al., 2019). However, many of these approaches focus on path planning or continuous-policy learning and do not directly address belief-aware corrective action refinement for trajectory tracking.

Tree search combined with learned value estimation is another important direction. MCTS-based approaches, such as AlphaGo, AlphaZero, and MuZero, and recent learned-model planning methods show that neural values or policy functions can guide search and improve action selection in complex sequential tasks (Silver et al., 2016; Silver et al., 2018; Schrittwieser et al., 2020; Danihelka et al., 2022). However, these methods are mainly demonstrated in games or general decision-making benchmarks and are not directly designed for deterministic solution-space best-first refinement in partially observable Mecanum robot tracking. Similarly, Alpha–Beta Minimax and AB–DDQN Hybrid provide adversarial lookahead, but their depth-first search may not always refine the most promising solution clusters first (Umakarthikeyan and Kumar, 2022; Ogunmolu and Gans, 2018; Isoyan, 2024; Wang et al., 2024b).

Recent hybrid search-learning and model-based reinforcement-learning methods further emphasize the importance of maintaining consistency between predicted internal states and observation-grounded states. Scholz et al. (2021) introduced reconstruction and consistency losses to constrain MuZero-style internal state predictions and stabilize learned model rollouts, while TD-MPC combines short-horizon model-predictive planning with a terminal value function and latent-state consistency learned through temporal-difference learning (Scholz et al., 2021; Hansen et al., 2022). Recent deep-learning and intelligent-perception studies have also addressed diverse tasks, including visual–textual diffusion-based underwater image enhancement (Wang et al., 2026), visual–textual mutual guidance for remote-sensing visual question answering (Liu et al., 2026), graph representation learning for plot-to-track association in compact HFSWR (Li et al., 2026), reinforcement-learning-based self-organized underwater image enhancement (Wang et al., 2024a), and smart underwater image-enhancement protocols (Wang et al., 2023). The proposed SSS*–DDQN is related to these works in the broad sense that it also maintains prediction consistency during lookahead-based action selection. However, it differs fundamentally because it does not learn an unconstrained latent dynamics model or optimize a self-supervised consistency loss. Instead, state consistency is maintained through EKF prediction and innovation-based correction using the nonlinear Mecanum robot model, measurement residual, and Kalman gain. The corrected belief mean and covariance are then used by QMDP sampling, DDQN value evaluation, and SSS* best-first refinement. Thus, the proposed method can be viewed as a physics-informed belief-consistent search-learning controller, rather than a latent-model self-supervised planning method.

To address these limitations, we propose an SSS*–DDQN solution-space best-first search controller for four-Mecanum-wheel robot trajectory tracking under partial observability. The proposed framework integrates EKF belief-state estimation, QMDP belief sampling, DDQN value evaluation, and SSS* OPEN-list search with LIVE/SOLVED node handling. It also uses beta-bound pruning, delayed MIN-sibling refinement, bounded OPEN-list management, and secondary lookahead to improve robust action selection while limiting unnecessary search expansion (An et al., 2026; Jestel et al., 2026; Tsai et al., 2026a; Slaoui, 2026).

The proposed method is compared with EKF-belief PER-DDQN, QMDP-PER-DDQN, Alpha–Beta Minimax, and AB–DDQN Hybrid. These baselines isolate the effects of belief-state value learning, belief sampling, adversarial search, and DDQN-guided search under the same partial-observability setting (Pham et al., 2025; Mehta et al., 2025; Jaramillo-Martínez and Chavero-Navarrete, 2026; Yu et al., 2023; Beinhofer et al., 2013). Although PPO, SAC, TD3, MPC, and standard MCTS are important modern comparators (An et al., 2026; Tsai et al., 2026a; Li et al., 2019; Li et al., 2024; Zhou et al., 2019), the present study focuses on controllers with the same discrete corrective-action and uncertainty-aware tracking structure to enable a controlled evaluation of the proposed SSS* best-first refinement.

The main research gap is that existing robust controllers improve stability but do not use learned value-guided search; belief-space RL methods handle uncertainty but do not refine future action clusters; Alpha–Beta search provides adversarial lookahead but is depth-first; and MCTS-based value-learning methods are not directly formulated for deterministic solution-space best-first refinement in partially observable Mecanum tracking. Therefore, this work contributes the following: (i) an uncertainty-aware SSS*–DDQN tracking framework, (ii) integration of EKF belief estimation and QMDP belief sampling, (iii) DDQN-guided SSS* best-first corrective action refinement, and (iv) comparative validation against four controlled baselines under noisy observations and sensor dropout.

The remainder of this paper is organized as follows. Section 2 presents the Mecanum robot model and partial-observability formulation. Section 3 describes the reinforcement-learning and baseline controllers. Section 4 introduces the proposed SSS*–DDQN framework. Section 5 presents simulation results and comparative analysis. Section 6 concludes the paper and discusses practical limitations and future work.

## 2 Mecanum robot modeling and partial-observability framework

The four-Mecanum-wheel mobile robot is modeled using its position, heading, and body-frame velocities to represent longitudinal, lateral, and rotational motion. The controller-generated force is distributed to the individual wheels through the Mecanum force-mapping mechanism with actuator saturation (Klančar et al., 2017; van der Spijk, 2024; Mao, 2025).

A lemniscate reference trajectory is used to evaluate omnidirectional tracking performance because it requires combined forward, sideways, and rotational motion. To represent practical sensing conditions, noisy measurements and occasional sensor dropout are included. An extended Kalman filter estimates the hidden robot state and maintains the belief mean and covariance, which are used by the reinforcement-learning and search-based controllers for robust action selection under uncertainty (Hendzel and Kołodziej, 2021; Ghiasi and Kiumarsi, 2025; Taheri and Xia, 2020; Tsai et al., 2026b; Lee, 2025).

Before introducing the mathematical model, the coordinate system, modeling assumptions, and control objectives of the four-Mecanum-wheel robot are defined. The inertial frame {I} represents the global reference frame, while the body-fixed frame {B} is attached to the geometric center of the robot. The robot posture is described by the inertial-frame position and heading angle, whereas the body-frame velocities describe longitudinal, lateral, and yaw motion. The robot is modeled as a planar rigid body moving on a flat surface. The wheel-ground contact is assumed to be non-slipping within the actuator saturation limits, and the four Mecanum wheels are independently driven. The main control objective is to make the robot track the desired lemniscate reference trajectory under noisy measurements, sensor dropout, bounded wheel forces, and partial observability. Therefore, the controller must minimize position error, heading error, control effort, and belief uncertainty while maintaining stable trajectory tracking.

### 2.1 Kinematic and dynamic model of the four-Mecanum-wheel robot

As shown in Figure 1, the four-Mecanum-wheel robot consists of a rectangular chassis with four independently driven wheels. The body frame is fixed at the robot center, where u , v , and r represent the longitudinal velocity, lateral velocity, and yaw rate, respectively. The inertial frame describes the global position and heading. The simulation uses 2l=0.50 m , 2d=0.40 m , a=0.05 m , m=10 kg , $I_{z}=0.50\;kgm^{2}$ , and $F_{\max}=18\;N$ per wheel (Klančar et al., 2017; van der Spijk, 2024).

**FIGURE 1:**
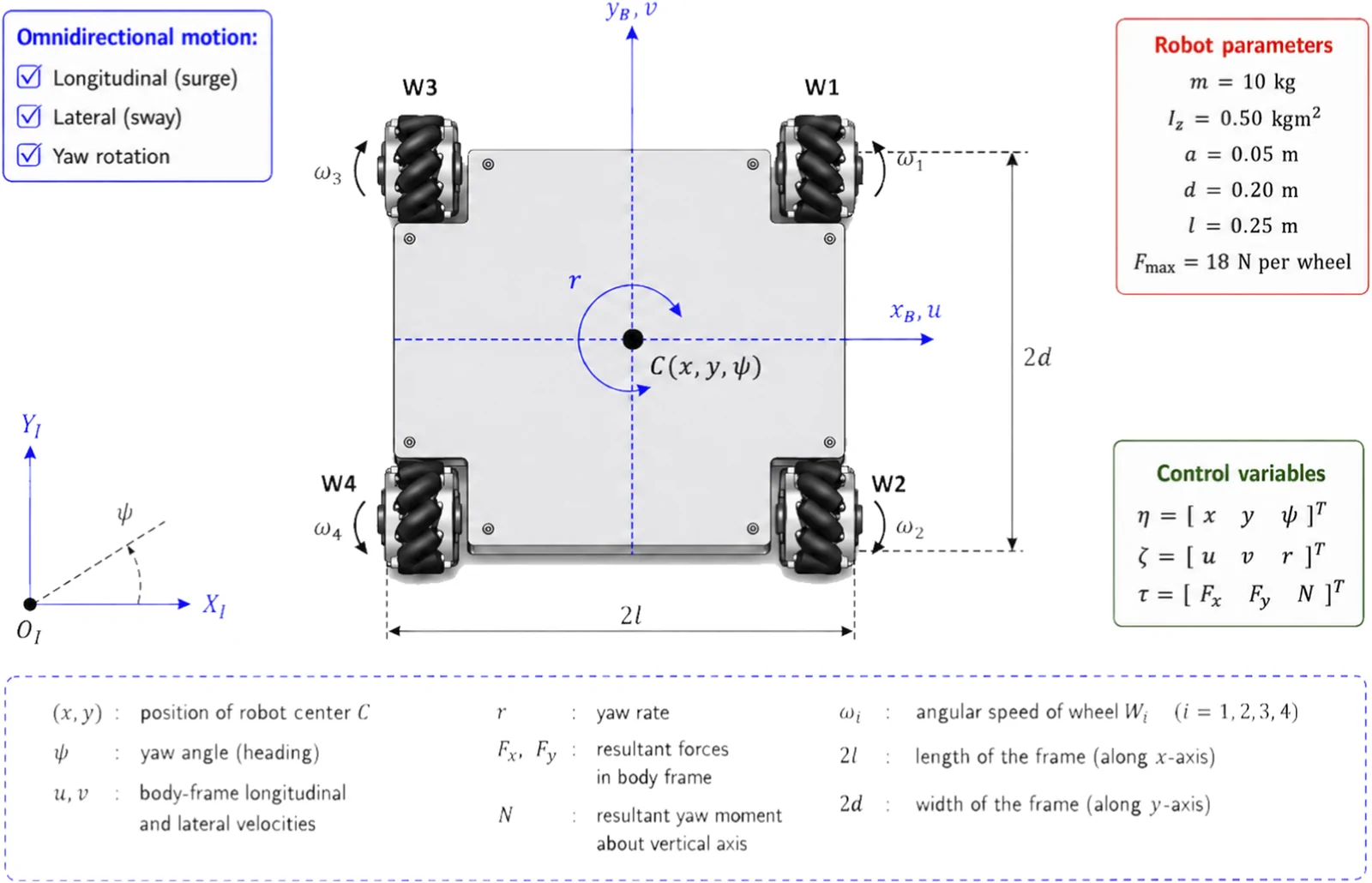
Four-Mecanum-wheel mobile robot geometry, body-frame, inertial-frame, wheel arrangement, and simulation parameters used for trajectory tracking analysis.

The robot posture and body-frame velocity are defined in Equations 1, 2, respectively:

$$\eta ={\left[\begin{array}{ccc} x & y & \psi \end{array}\right]}^{\text{T}},$$ (1)

$$\zeta ={\left[\begin{array}{ccc} u & v & r \end{array}\right]}^{\text{T}}.$$ (2)

Here, x and y are the inertial-frame position, ψ is the heading angle, and u , v , and r are the body-frame velocities.

The kinematic relation is given in Equation 3:

$$\dot{\eta }=J\left(\psi \right)\zeta ,$$ (3)

where (Equation 4)

$$J\left(\psi \right)=\left[\begin{array}{ccc} \cos\psi & -\sin\psi & 0\\ \sin\psi & \cos\psi & 0\\ 0 & 0 & 1 \end{array}\right].$$ (4)

This transformation converts body-frame motion into inertial-frame motion for trajectory tracking.

The dynamic model is expressed in Equation 5:

$$D\dot{\zeta }+n\left(\zeta \right)+B_{\text{v}}\zeta =\tau ,$$ (5)

where D is the inertia matrix, n(ζ) is the nonlinear velocity-coupling term, $B_{v}$ is the damping matrix, and τ is the generalized control force. These terms are defined in Equations 6–8:

$$D=\left[\begin{array}{ccc} m & 0 & 0\\ 0 & m & 0\\ 0 & 0 & I_{z} \end{array}\right],$$ (6)

$$n\left(\zeta \right)=\left[\begin{array}{c} -mrv\\ mru\\ 0 \end{array}\right],$$ (7)

and

$$B_{\text{v}}=\left[\begin{array}{ccc} 2.8 & 0 & 0\\ 0 & 2.8 & 0\\ 0 & 0 & 0.50 \end{array}\right].$$ (8)

The dynamic model represents the force–motion behavior of the Mecanum robot, including inertia, velocity coupling, and damping effects (Pandey and Gajbhiye, 2024; Anil et al., 2025; Banik et al., 2025; Chen, 2024; Shahgholian et al., 2024).

The simulation uses m=10 kg , $I_{z}=0.50\;kgm^{2}$ , a=0.05 m , d=0.20 m , and l=0.25 m . The body-frame acceleration is obtained as shown in Equation 9:

$$\dot{\zeta }=D^{-1}\left[\tau -n\left(\zeta \right)-B_{\text{v}}\zeta \right].$$ (9)

The velocity and posture are updated using Euler integration, as shown in Equations 10, 11, respectively:

$$\zeta_{k+1}=\zeta_{k}+\Delta t\;{\dot{\zeta }}_{k},$$ (10)

$$\eta_{k+1}=\eta_{k}+\Delta t\;J\left(\psi_{k}\right)\zeta_{k},$$ (11)

where Δt=0.02 s . This update scheme is simple and computationally efficient for repeated reinforcement-learning and search-based trajectory simulations.

### 2.2 Numerical integration fidelity and rk4 sensitivity check

To examine whether the use of Euler integration affects the comparative conclusions, a numerical-integration sensitivity check was performed using the fourth-order Runge–Kutta method. The RK4 update was used only as a verification step, while the main controller comparison retained Euler integration for computational efficiency and consistency with the discrete-time control implementation. For the robot dynamics presented in Equation 9, the RK4 update can be expressed as shown in Equation 12:

$$\zeta_{k+1}=\zeta_{k}+\frac{\Delta t}{6}\left(k_{1}+2k_{2}+2k_{3}+k_{4}\right),$$ (12)

where (Equations 13–16)

$$k_{1}=f\left(\zeta_{k},\tau_{k}\right),$$ (13)

$$k_{2}=f\left(\zeta_{k}+\frac{\Delta t}{2}k_{1},\tau_{k}\right),$$ (14)

$$k_{3}=f\left(\zeta_{k}+\frac{\Delta t}{2}k_{2},\tau_{k}\right),$$ (15)

and

$$k_{4}=f\left(\zeta_{k}+\Delta t\;k_{3},\tau_{k}\right).$$ (16)

The posture update is then computed using the updated body-frame velocity and the kinematic transformation matrix J(ψ) . The RK4 sensitivity check was used to verify that the relative controller behavior and overall ranking trend are not caused by the numerical integration method. Since all controllers are evaluated under identical numerical and sensing conditions, the reported comparative improvement in the proposed SSS*–DDQN controller remains a controller-level effect rather than an artifact of the Euler discretization.

### 2.3 Control force, wheel velocity, and lemniscate reference mapping

The generalized control force is defined in Equation 17:

$$\tau ={\left[\begin{array}{ccc} F_{x} & F_{y} & N \end{array}\right]}^{\text{T}},$$ (17)

where $F_{x}$ , $F_{y}$ , and N denote longitudinal force, lateral force, and yaw moment, respectively. This force is distributed to the four Mecanum wheels as shown in Equation 18:

τ=Γκ, (18)

where (Equations 19, 20)

$$\kappa ={\left[\begin{array}{cccc} F_{1} & F_{2} & F_{3} & F_{4} \end{array}\right]}^{\text{T}},$$ (19)

and

$$\Gamma =\frac{1}{\sqrt{2}}\left[\begin{array}{cccc} 1 & 1 & 1 & 1\\ 1 & -1 & 1 & -1\\ -\left(d+l\right) & -\left(d+l\right) & d+l & d+l \end{array}\right],$$ (20)

The wheel forces are obtained using Equation 21,

$$\kappa =\Gamma^{†}\tau_{\text{cmd}},$$ (21)

and limited by Equation 22:

$$-F_{\text{max}}\leq F_{i}\leq F_{\text{max}},\;i=1,2,3,4,$$ (22)

where $F_{\max}=18\;N$ . This mapping converts the controller command into feasible wheel-level forces.

The body-frame velocity and wheel angular velocity are related by Equation 23:

ζ=Wω, (23)

where (Equations 24, 25)

$$\omega ={\left[\begin{array}{cccc} \omega_{1} & \omega_{2} & \omega_{3} & \omega_{4} \end{array}\right]}^{\text{T}},$$ (24)

and

$$W=\frac{a}{4}\left[\begin{array}{cccc} 1 & 1 & 1 & 1\\ 1 & -1 & 1 & -1\\ -\frac{1}{d+l} & -\frac{1}{d+l} & \frac{1}{d+l} & \frac{1}{d+l} \end{array}\right],$$ (25)

This relation verifies that the selected control action produces feasible longitudinal, lateral, and rotational motion through coordinated wheel velocities (Klančar et al., 2017; van der Spijk, 2024; Mao, 2025).

A lemniscate reference trajectory is used to evaluate omnidirectional tracking performance. The desired trajectory is defined in Equations 26, 27:

$$x_{\text{d}}\left(t\right)=A\sin\left(\omega_{\text{r}}t\right),$$ (26)

$$y_{\text{d}}\left(t\right)=A\sin\left(\omega_{\text{r}}t\right)\cos\left(\omega_{\text{r}}t\right),$$ (27)

where A=1.5 and $\omega_{r}=0.35\;rad/s$ . The reference velocities are presented in Equations 28, 29:

$${\dot{x}}_{\text{d}}\left(t\right)=A\omega_{\text{r}}\cos\left(\omega_{\text{r}}t\right),$$ (28)

$${\dot{y}}_{\text{d}}\left(t\right)=A\omega_{\text{r}}\cos\left(2\omega_{\text{r}}t\right).$$ (29)

The desired heading is obtained from the reference velocity direction, as shown in Equation 30:

$$\psi_{\text{d}}\left(t\right)=\text{atan}2\;\left({\dot{y}}_{\text{d}}\left(t\right),{\dot{x}}_{\text{d}}\left(t\right)\right).$$ (30)

The yaw-rate and yaw-acceleration references are computed numerically as shown in Equations 31, 32:

$$r_{\text{d}}\left(t\right)=\frac{d\psi_{\text{d}}\left(t\right)}{dt},$$ (31)

$${\dot{r}}_{\text{d}}\left(t\right)=\frac{dr_{\text{d}}\left(t\right)}{dt}.$$ (32)

The lemniscate path is selected because it continuously changes curvature and requires combined longitudinal, lateral, and rotational motion. Therefore, it is suitable for testing Mecanum robot tracking capability (Pandey and Gajbhiye, 2024; Anil et al., 2025; Banik et al., 2025; Chen, 2024; Shahgholian et al., 2024).

The posture error is transformed into the body frame as shown in Equation 33:

$$e_{\text{b}}=J^{\text{T}}\left(\psi \right)\left(\eta_{\text{d}}-\eta \right).$$ (33)

The desired and commanded body-frame velocities are presented in Equations 34, 35:

$$\zeta_{\text{d}}=J^{\text{T}}\left(\psi \right){\dot{\eta }}_{\text{d}},$$ (34)

$$\zeta_{\text{cmd}}=\zeta_{\text{d}}+K_{\text{p}}e_{\text{b}}.$$ (35)

The nominal torque command is computed as shown in Equation 36:

$$\tau_{\text{nom}}=D{\dot{\zeta }}_{\text{cmd}}+n\left(\zeta \right)+B_{\text{v}}\zeta +K_{\tau }\left(\zeta_{\text{cmd}}-\zeta \right).$$ (36)

This nominal controller provides model-based tracking compensation, while reinforcement-learning and search-based methods add corrective actions to improve robustness under uncertainty (Saidi and Telmoudi, 2026; Ben Hazem et al., 2025; López-González et al., 2025; Vazquez-Santiago, 2025).

The robot parameters, controller gains, and simulation constants used in Mecanum robot model are summarized in Table 1. These values define the physical robot structure, actuator force limit, reference trajectory settings, and control gains used for trajectory tracking simulation.

**TABLE 1:** Robot parameters, control gains, and simulation constants used in the Mecanum robot model.

Parameter	Symbol	Value
Robot mass	m	10 kg
Yaw moment of inertia	$I_{z}$	$0.50\;kg\;m^{2}$
Wheel radius	a	0.05 m
Half-width of robot	d	0.20 m
Half-length of robot	l	0.25 m
Robot width	2d	0.40 m
Robot length	2l	0.50 m
Maximum wheel force	$F_{\text{max}}$	18 N per wheel
Sampling time	Δt	0.02 s
Total simulation time	$T_{\text{f}}$	20 s
Reference trajectory amplitude	A	1.5 m
Reference angular frequency	$\omega_{\text{r}}$	$0.35\;rad\;s^{-1}$
Position control gain	$K_{\text{p}}$	diag (14,14,10)
Velocity damping gain	$K_{\text{d}}$	diag (8,8,5)
Torque correction gain	$K_{\tau }$	diag (10,10,6)
Viscous damping matrix	$B_{\text{v}}$	diag (2.8,2.8,0.50)

### 2.4 Noisy observation model and sensor dropout

The robot is simulated under partial observability, where the true state is not directly available to the controller. Instead, noisy measurements of position, heading, body-frame velocity, and yaw rate are used, as shown in Equations 37, 38:

$$x_{k}={\left[\begin{array}{cccccc} x_{k} & y_{k} & \psi_{k} & u_{k} & v_{k} & r_{k} \end{array}\right]}^{\text{T}}.$$ (37)

$$z_{k}=x_{k}+\nu_{k}.$$ (38)

where $\nu_{k}$ is the zero-mean sensor noise.

The measurement-noise covariance is presented in Equation 39:

$$R=\text{diag }\left(\sigma_{\text{p}}^{2},\sigma_{\text{p}}^{2},\sigma_{\psi }^{2},\sigma_{\text{v}}^{2},\sigma_{\text{v}}^{2},\sigma_{\text{r}}^{2}\right).$$ (39)

with Equation 40:

$$\sigma_{\text{p}}=0.035\;m,\;\sigma_{\psi }=1.5{}^{\circ},\;\sigma_{\text{v}}=0.035\;m\;s^{-1},\;\sigma_{\text{r}}=1.2{}^{\circ}s^{-1}.$$ (40)

To model temporary localization degradation, position dropout is included with Equation 41:

$$P_{\text{drop}}=0.03.$$ (41)

During dropout, the position measurement is corrupted as shown in Equation 42:

$$z_{k}\left(1:2\right)=x_{k}\left(1:2\right)+4\sigma_{\text{p}}N\left(0,I_{2}\right).$$ (42)

This observation model represents practical sensing uncertainty and supports EKF-based belief estimation for robust control.

Sensor dropout represents temporary localization loss due to wheel slip, occlusion, communication delay, or sensor disturbance. Therefore, the controller requires a belief-state representation instead of relying only on noisy measurements (Hendzel and Kołodziej, 2021; Ghiasi and Kiumarsi, 2025; Taheri and Xia, 2020; Tsai et al., 2026b; Lee, 2025).

The noisy observation and dropout model justify the use of an EKF to estimate the hidden robot state and belief covariance. The sensor-noise, dropout, and EKF covariance settings are summarized in Table 2. These settings support robust action selection under partial observability (Tsai et al., 2026b; Beinhofer et al., 2013; Yao et al., 2024; Yao et al., 2025b; Yao et al., 2025a).

**TABLE 2:** Sensor-noise, dropout-probability, and EKF covariance settings used in the partial-observability model.

Parameter	Symbol	Value
Position measurement noise	$\sigma_{\text{p}}$	0.035 m
Heading measurement noise	$\sigma_{\psi }$	1.5°
Velocity measurement noise	$\sigma_{\text{v}}$	$0.035\;m\;s^{-1}$
Yaw-rate measurement noise	$\sigma_{\text{r}}$	$1.2{}^{\circ}s^{-1}$
Sensor dropout probability	$P_{\text{drop}}$	0.03.
Measurement covariance matrix	R.	$\text{diag}\;(\sigma_{\text{p}}^{2},\sigma_{\text{p}}^{2},\sigma_{\psi }^{2},$ $\sigma_{\text{v}}^{2},\sigma_{\text{v}}^{2},\sigma_{\text{r}}^{2})$
Initial position covariance	$P_{0,x},\;P_{0,y}$	$0.10^{2}$
Initial heading covariance	$P_{0,\psi }$	${\left(5{}^{\circ}\right)}^{2}$
Initial velocity covariance	$P_{0,u},\;P_{0,v}$	$0.08^{2}$
Initial yaw-rate covariance	$P_{0,r}$	${\left(3{}^{\circ}\right)}^{2}$
EKF process noise for position	$Q_{x},\;Q_{y}$	$1\times 10^{-4}$
EKF process noise for heading	$Q_{\psi }$	$1\times 10^{-5}$
EKF process noise for velocity	$Q_{u},\;Q_{v}$	$2\times 10^{-3}$
EKF process noise for the yaw rate	$Q_{r}$	$8\times 10^{-4}$

### 2.5 EKF-based belief-state estimation

To address partial observability, an EKF estimates the hidden robot state using noisy sensor measurements. The EKF belief state is represented by the estimated mean ${\hat{x}}_{k}$ and covariance $P_{k}$ , where the covariance indicates state-estimation uncertainty (Taheri and Xia, 2020; Tsai et al., 2026b).

The EKF is initialized as shown in Equations 43, 44:

$${\hat{x}}_{0}=z_{0}.$$ (43)

$$P_{0}=\text{diag }\left(0.10^{2},0.10^{2},{\left(5{}^{\circ}\right)}^{2},0.08^{2},0.08^{2},{\left(3{}^{\circ}\right)}^{2}\right).$$ (44)

During prediction, the nonlinear Mecanum robot model estimates the next belief state as shown in Equations 45, 46:

$${\hat{x}}_{k|k-1}=f\left({\hat{x}}_{k-1},\tau_{k}\right),$$ (45)

$$P_{k|k-1}=F_{k}P_{k-1}F_{k}^{\text{T}}+Q,$$ (46)

where $F_{k}$ is the numerically computed state-transition Jacobian and Q is the process-noise covariance (Equation 47):

$$Q=\text{diag }\left(10^{-4},10^{-4},10^{-5},2\times 10^{-3},2\times 10^{-3},8\times 10^{-4}\right).$$ (47)

Since all six state variables are measured with noise, the observation matrix is presented in Equation 48:

$$H=I_{6}.$$ (48)

The EKF correction step is provided in Equations 49–53:

$$y_{k}=z_{k}-H{\hat{x}}_{k|k-1},$$ (49)

$$S_{k}=HP_{k|k-1}H^{\text{T}}+R,$$ (50)

$$K_{k}=P_{k|k-1}H^{\text{T}}S_{k}^{-1},$$ (51)

$${\hat{x}}_{k}={\hat{x}}_{k|k-1}+K_{k}y_{k},$$ (52)

$$P_{k}=\left(I_{6}-K_{k}H\right)P_{k|k-1}{\left(I_{6}-K_{k}H\right)}^{\text{T}}+K_{k}RK_{k}^{\text{T}}.$$ (53)

The Joseph covariance update improves numerical stability and preserves a valid covariance matrix. The resulting belief mean and covariance are used by QMDP, Alpha–Beta, AB–DDQN, and SSS*–DDQN controllers for uncertainty-aware action selection (Hendzel and Kołodziej, 2021; Ghiasi and Kiumarsi, 2025; Tsai et al., 2026b; Lee, 2025).

As shown in the flowchart in Figure 2, the EKF belief update consists of prediction and correction stages. The prediction stage uses the previous belief and control input with the nonlinear robot model to estimate the next state and covariance. The correction stage uses noisy measurements to update the belief mean and covariance through innovation and Kalman gain. Thus, the EKF converts uncertain sensor data into a reliable belief-state representation, enabling robust controller decisions under partial observability.

**FIGURE 2:**
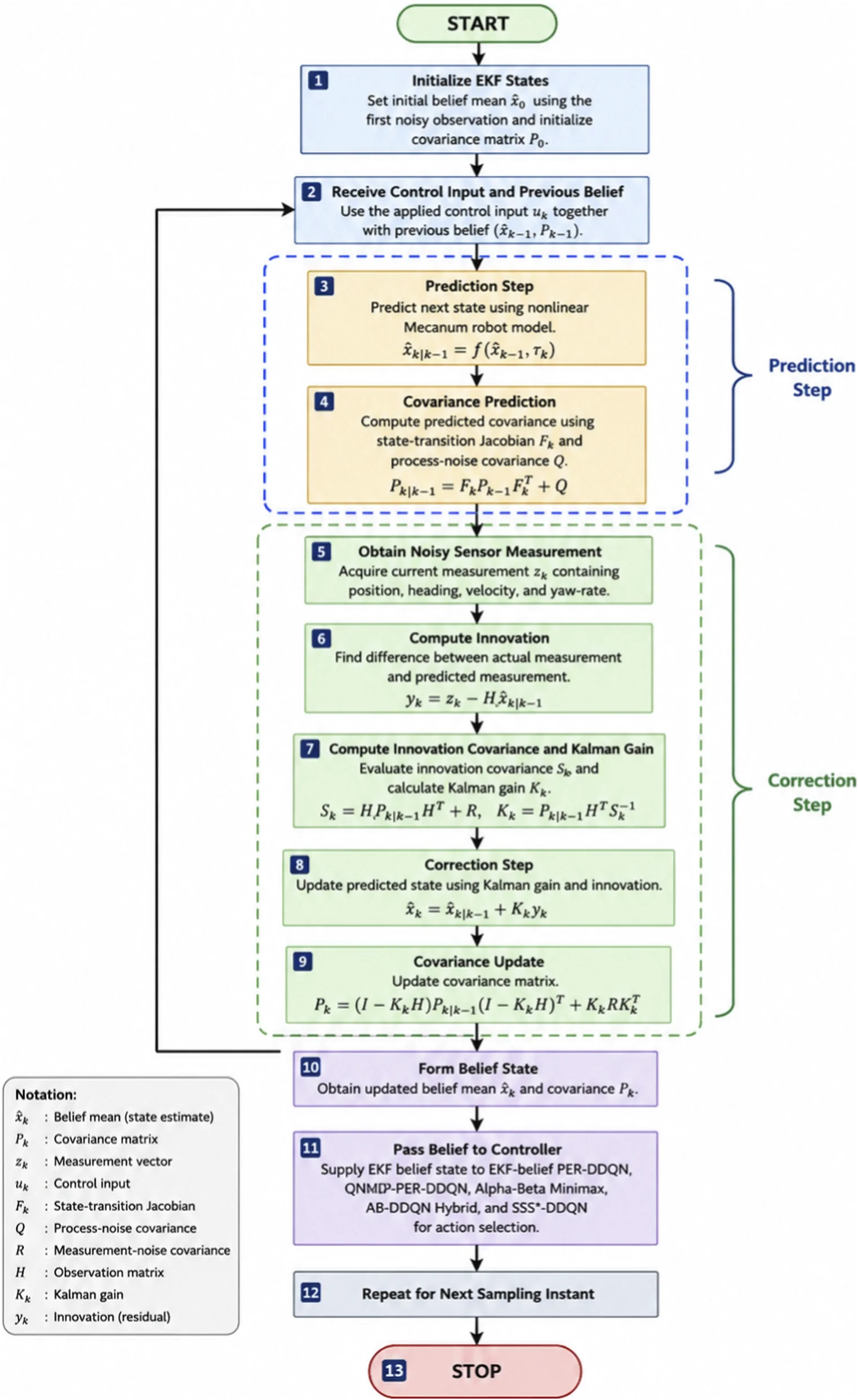
EKF prediction–correction belief update process used to estimate the hidden Mecanum robot state from noisy measurements and provide belief information to the control algorithms.

## 3 Reinforcement learning and baseline control methods

This section presents the baseline controllers used to evaluate the proposed SSS*–DDQN method. Four methods are considered: EKF-belief PER-DDQN, QMDP-PER-DDQN, Alpha–Beta Minimax, and AB–DDQN Hybrid. These baselines compare learning-based control, belief-state control, adversarial search, and hybrid search-learning control under the same Mecanum robot tracking condition (Umakarthikeyan and Kumar, 2022; Ogunmolu and Gans, 2018; Isoyan, 2024; Wang et al., 2024).

PER-DDQN improves value learning using prioritized replay and reduced overestimation. EKF-belief PER-DDQN uses filtered belief states, while QMDP-PER-DDQN evaluates actions over belief samples. Alpha–Beta Minimax considers worst-case disturbances, and AB–DDQN Hybrid combines Alpha–Beta search with DDQN leaf evaluation. Thus, these baselines provide a strong comparison for the proposed SSS*–DDQN controller (An et al., 2026; Jestel et al., 2026; Tsai et al., 2026a; Slaoui, 2026).

### 3.1 PER-DDQN formulation with state, action, and reward design

The PER-DDQN controller is designed to learn corrective actions for the Mecanum robot while reducing Q-value overestimation and improving training efficiency through prioritized experience replay. The learned action is added to the nominal model-based torque command, allowing the controller to combine model-based compensation with reinforcement-learning adaptation (Wang et al., 2024b; An et al., 2026; Jestel et al., 2026; Tsai et al., 2026a; Slaoui, 2026).

This 15-dimensional state vector provides both current tracking information and reference trajectory information, enabling the agent to select suitable corrective actions under uncertainty (Pham et al., 2025; Mehta et al., 2025; Jaramillo-Martínez and Chavero-Navarrete, 2026; Yu et al., 2023).

The action space is defined using longitudinal, lateral, and yaw corrective components, as shown in Equation 54:

$$\Delta F_{x}\in \left\{-3,0,3\right\},\;\Delta F_{y}\in \left\{-3,0,3\right\},\;\Delta N\in \left\{-1,0,1\right\}.$$ (54)

The discrete action vector is presented in Equation 55:

$$a_{k}={\left[\begin{array}{ccc} \Delta F_{x} & \Delta F_{y} & \Delta N \end{array}\right]}^{\text{T}}.$$ (55)

Thus, the total number of actions is shown in Equation 56:

$$N_{\text{a}}=3\times 3\times 3=27.$$ (56)

This action design keeps online selection simple while allowing correction in the three controllable motion directions of the Mecanum robot. The selected corrective action is added to the nominal torque command to improve tracking without replacing the model-based controller.

During training, the control input is computed as shown in Equation 57:

$$\tau_{\text{cmd}}=0.75\tau_{\text{nom}}+a_{k},$$ (57)

where $\tau_{\text{nom}}$ is the nominal torque and $a_{k}$ is the DDQN corrective action. This blended command improves stability by combining model-based tracking with learned correction.

The reward function penalizes position error, heading error, velocity error, control effort, and actuator saturation, as shown in Equation 58:

$$\begin{array}{cc} R_{k}= & -18.0e_{xy}^{2}-3.5e_{\psi }^{2}-0.30{\left\|\zeta_{\text{d}}-\zeta \right\|}_{2}^{2}\\ & -0.015{\left\|a_{k}\right\|}_{2}^{2}-0.20P_{\text{sat}}. \end{array}$$ (58)

A bonus is added for accurate tracking, as presented in Equation 59:

$$R_{k}=R_{k}+2.0,\;if\;e_{xy}<0.05\;and\;\left|e_{\psi }\right|<0.05.$$ (59)

The episode is terminated under large tracking or heading error, as shown in Equation 60:

$$e_{xy}>3.0\;or\;\left|e_{\psi }\right|>0.95\pi .$$ (60)

Prioritized experience replay stores transitions and samples important experiences using temporal-difference errors. The double-DQN target is presented in Equation 61:

$$\begin{array}{cc} Y_{k}= & R_{k}+\gamma \left(1-d_{k}\right)Q_{\text{target}}\left(s_{k+1},\right.\underset{a\in A}{arg\;max}Q_{\text{online}}\left.\left(s_{k+1},a\right)\right). \end{array}$$ (61)

This reduces overestimation by selecting the next action with the online network and evaluating it with the target network.

The main RL parameters are summarized in Table 3. Overall, the 15-dimensional state, 27-action space, and reward design provide a stable PER-DDQN formulation for robust Mecanum robot trajectory tracking under uncertainty. The DDQN network architecture consists of a 15-dimensional input layer, three fully connected hidden layers with 192, 192, and 128 neurons, and a 27-dimensional output layer corresponding to the discrete corrective-action set. Rectified linear unit (ReLU) activation functions are used after each hidden layer to improve nonlinear feature representation and training stability. The output layer uses a linear activation function to directly estimate the Q-value of each discrete action. To support learning stability, the training convergence was monitored using cumulative reward, temporal-difference loss, exploration-rate decay, and tracking-error reduction. The cumulative reward improves progressively, the loss decreases and stabilizes, and the exploration rate decays smoothly from the initial value to the final value. These trends confirm that the DDQN training process remains stable before deterministic controller evaluation.

**TABLE 3:** RL state vector, action set, reward terms, and hyperparameters used in the PER-DDQN formulation.

Category	Symbol/Term	Description/Value
State vector dimension	$n_{\text{s}}$	15
Tracking error states	$e_{x},\;e_{y},\;e_{\psi }$	Position and heading errors
Robot velocity states	u, v, r	Body-frame longitudinal, lateral, and yaw velocities
Error-rate states	${\dot{e}}_{x},\;{\dot{e}}_{y},\;{\dot{e}}_{\psi }$	Rates of tracking-error variation
Reference velocity states	$u_{\text{d}},\;v_{\text{d}},\;r_{\text{d}}$	Desired body-frame velocity components
Reference posture states	$x_{\text{d}},\;y_{\text{d}},\;\psi_{\text{d}}$	Desired position and heading angle
Action vector	$a_{k}$	${\left[\Delta F_{x},\Delta F_{y},\Delta N\right]}^{\text{T}}$
Longitudinal force action	$\Delta F_{x}$	{−3,0,3}
Lateral force action	$\Delta F_{y}$	{−3,0,3}
Yaw-moment action	ΔN	{−1,0,1}
Total number of actions	$N_{\text{a}}$	27
Control command	$\tau_{\text{cmd}}$	$0.75\tau_{\text{nom}}+a_{k}$
Position-error reward weight	$w_{\text{p}}$	18.0
Heading-error reward weight	$w_{\psi }$	3.5
Velocity-error reward weight	$w_{\text{v}}$	0.30
Control-effort penalty weight	$w_{\text{a}}$	0.015
Saturation penalty weight	$w_{\text{s}}$	0.20
Tracking bonus	$R_{\text{bonus}}$	+2.0 if $e_{xy}<0.05$ and $|e_{\psi }|<0.05$
Failure penalty	$R_{\text{fail}}$	−50
Termination condition	–	$e_{xy}>3.0$ or $|e_{\psi }|>0.95\pi$
Discount factor	γ	0.985
Learning rate	α	$1\times 10^{-4}$
Batch size	–	64
Replay-memory capacity	–	60,000
Training start step	–	1,200
Training interval	–	Every four steps
Target soft-update factor	$\tau_{\text{target}}$	0.005
Huber-loss threshold	–	1.0
Gradient-clipping value	–	1.0
Initial exploration rate	$\epsilon_{\text{start}}$	1.0
Final exploration rate	$\epsilon_{\text{end}}$	0.05
Exploration decay factor	–	0.992
PER priority exponent	$\alpha_{\text{PER}}$	0.6
Initial importance-sampling factor	$\beta_{0}$	0.4
Final importance-sampling factor	$\beta_{\text{end}}$	1.0
PER priority offset	$\epsilon_{\text{PER}}$	$1\times 10^{-3}$
Priority-clipping value	–	10.0
QMDP belief samples	$N_{\text{s}}$	15
Network hidden layers	–	192–192–128 neurons
Network output size	$N_{\text{a}}$	27 Q-values

### 3.2 EKF-belief PER-DDQN controller

The EKF-belief PER-DDQN controller uses the EKF-estimated belief state for action selection instead of the unavailable true state. At each sampling instant, the EKF provides the estimated state as shown in Equation 62:

$${\hat{x}}_{k}={\left[\begin{array}{cccccc} {\hat{x}}_{k} & {\hat{y}}_{k} & {\hat{\psi }}_{k} & {\hat{u}}_{k} & {\hat{v}}_{k} & {\hat{r}}_{k} \end{array}\right]}^{\text{T}}.$$ (62)

The normalized DDQN input is constructed from the EKF belief state, reference trajectory, and previous tracking error as shown in Equation 63:

$$s_{k}^{\text{EKF}}=\frac{\Phi \left({\hat{x}}_{k},\eta_{\text{d},k},{\dot{\eta }}_{\text{d},k},e_{k-1}\right)}{s_{\text{scale}}}.$$ (63)

The corrective action is selected using the trained DDQN network, as shown in Equation 64:

$$a_{k}=\underset{a\in A}{arg\;max}Q_{\theta }\left(s_{k}^{\text{EKF}},a\right).$$ (64)

During training, the control command is presented in Equation 65:

$$\tau_{\text{cmd}}=0.75\tau_{\text{nom}}+a_{k},$$ (65)

whereas during deterministic evaluation, it is presented in Equation 66:

$$\tau_{\text{cmd}}=\tau_{\text{nom}}+a_{k}.$$ (66)

Thus, the nominal controller provides model-based tracking, while the DDQN supplies adaptive corrective action. Algorithm 1 summarizes this closed-loop belief-update and action-selection process for robust tracking under noisy and partially observable conditions (Hendzel and Kołodziej, 2021; Ghiasi and Kiumarsi, 2025; Taheri and Xia, 2020; Tsai et al., 2026b; Lee, 2025).

Algorithm 1 EKF-belief PER-DDQN action-selection procedure.

1. Input: Noisy observation $z_{k}$ , previous belief $\left({\hat{x}}_{k-1},P_{k-1}\right)$ , reference trajectory $\left(\eta_{d,k},{\dot{\eta }}_{d,k},{\ddot{\eta }}_{d,k}\right)$ , trained DDQN network $Q_{\theta }$ , and action set A .
2. Output: Control command $\tau_{\text{cmd}}$ .
3. Initialize EKF belief state $\left({\hat{x}}_{0},P_{0}\right)$ using the first noisy observation.
4. For each sampling instant k :
  - Predict the belief mean using the nonlinear Mecanum robot model:
$${\hat{x}}_{k|k-1}=f\left({\hat{x}}_{k-1},\tau_{k-1}\right)$$
  - Predict the belief covariance:
$$P_{k|k-1}=F_{k}P_{k-1}F_{k}^{T}+Q$$
  - Receive noisy sensor measurement $z_{k}$ .
  - Compute innovation:
$$y_{k}=z_{k}-H{\hat{x}}_{k|k-1}$$
  - Compute Kalman gain:
$$K_{k}=P_{k|k-1}H^{T}{\left(HP_{k|k-1}H^{T}+R\right)}^{-1}$$
  - Correct the belief mean:
$${\hat{x}}_{k}={\hat{x}}_{k|k-1}+K_{k}y_{k}$$
  - Correct the belief covariance:
$$P_{k}=\left(I-K_{k}H\right)P_{k|k-1}{\left(I-K_{k}H\right)}^{T}+K_{k}RK_{k}^{T}$$
  - Construct and normalize the DDQN input state:
$${\bar{s}}_{k}^{\text{EKF}}=\frac{\Phi \left({\hat{x}}_{k},\eta_{d,k},{\dot{\eta }}_{d,k},e_{k-1}\right)}{s_{\text{scale}}}$$
  - Evaluate Q-values for all discrete actions:
$$Q_{k}=Q_{\theta }\left({\bar{s}}_{k}^{\text{EKF}},a\right),\;a\in A$$
  - Select the best corrective action:
$$a_{k}=\arg\underset{a\in A}{\max}Q_{\theta }\left({\bar{s}}_{k}^{\text{EKF}},a\right)$$
  - Compute nominal torque $\tau_{\text{nom}}$ and final command:
$$\tau_{\text{cmd}}=\tau_{\text{nom}}+a_{k}$$
  - Apply $\tau_{\text{cmd}}$ to the Mecanum robot.
  - Store updated belief $\left({\hat{x}}_{k},P_{k}\right)$ for the next sampling instant.

### 3.3 QMDP-PER-DDQN with belief sampling

The QMDP-PER-DDQN controller improves EKF-belief PER-DDQN by evaluating actions over multiple belief samples instead of using only the EKF mean. The EKF covariance is first stabilized as shown in Equation 67:

$$P_{k}^{\text{s}}=\frac{P_{k}+P_{k}^{\text{T}}}{2}+\epsilon I_{6},$$ (67)

and belief samples are generated as presented in Equation 68:

$$x_{k}^{\left(i\right)}={\hat{x}}_{k}+L_{k}\xi^{\left(i\right)},\;i=1,2,\ldots ,N_{\text{s}},$$ (68)

where $N_{s}=15$ .

For each belief sample, the normalized DDQN input is constructed as shown in Equation 69:

$${\bar{s}}_{k}^{\left(i\right)}=\frac{\Phi \left(x_{k}^{\left(i\right)},\eta_{\text{d},k},{\dot{\eta }}_{\text{d},k},e_{k-1}\right)}{s_{\text{scale}}},$$ (69)

and the Q-values are evaluated using Equation 70:

$$Q^{\left(i\right)}\left(a\right)=Q_{\theta }\left({\bar{s}}_{k}^{\left(i\right)},a\right),\;a\in A.$$ (70)

The averaged Q-value is computed using Equation 71:

$$\bar{Q}\left(a\right)=\frac{1}{N_{\text{s}}}\overset{N_{\text{s}}}{\underset{i=1}{\sum }}Q_{\theta }\left({\bar{s}}_{k}^{\left(i\right)},a\right),$$ (71)

and the best action is selected using Equation 72:

$$a_{k}=\underset{a\in A}{arg\;max}\bar{Q}\left(a\right).$$ (72)

The final control command is presented in Equation 73:

$$\tau_{\text{cmd}}=\tau_{\text{nom}}+a_{k}.$$ (73)

QMDP belief sampling improves robustness by considering multiple possible hidden states under sensor noise and dropout (Tsai et al., 2026b; Beinhofer et al., 2013; Yao et al., 2024; Yao et al., 2025b; Yao et al., 2025a). Algorithm 2 summarizes this uncertainty-aware action evaluation procedure, where averaged DDQN Q-values are used to select a reliable corrective action for comparison with the proposed SSS*–DDQN controller (Wang et al., 2024b; An et al., 2026; Jestel et al., 2026; Li et al., 2019; Li et al., 2024).

Algorithm 2 QMDP belief-sampling-based action evaluation.

1. Input: EKF belief mean, EKF covariance, trained DDQN network, action set, reference trajectory, previous tracking error, and state-scaling vector.
2. Symmetrize the EKF covariance matrix to avoid numerical instability.
3. Generate belief samples around the EKF mean using the covariance matrix.
4. For each belief sample:
  - Extract sampled position, heading, velocity, and yaw-rate information.
  - Construct the DDQN input state using sampled belief state, tracking error, reference velocity, and reference posture.
  - Normalize the input state using the state-scaling vector.
  - Evaluate Q-values for all discrete actions using the trained DDQN network.
5. Average the Q-values of each action over all belief samples.
6. Select the action with the highest averaged Q-value.
7. Compute the nominal model-based torque command.
8. Add the selected QMDP-DDQN corrective action to the nominal torque command.
9. Apply the final control command to the Mecanum robot.
10. Output: Selected robust action and final control command.

### 3.4 Alpha–Beta Minimax and AB–DDQN Hybrid controllers

The Alpha–Beta Minimax controller is used as a search-based baseline under partial observability. As shown in Figure 3, it uses a three-ply MAX–MIN–MAX structure, where MAX nodes represent robot corrective-action selection and MIN nodes represent worst-case disturbance or uncertainty. This allows the controller to select actions that remain effective under noisy and uncertain conditions (Umakarthikeyan and Kumar, 2022; Ogunmolu and Gans, 2018; Pham et al., 2025).

**FIGURE 3:**
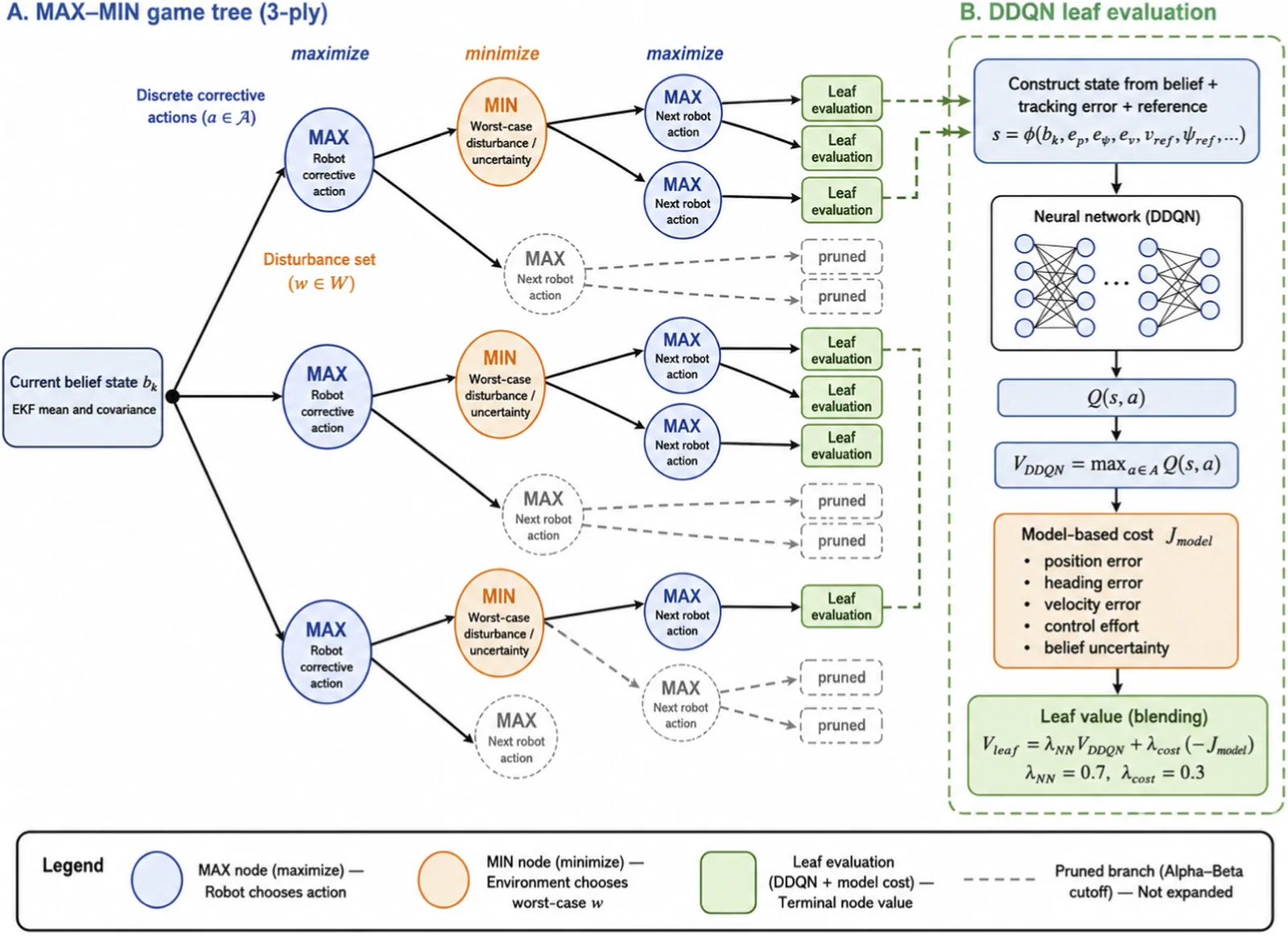
MAX–MIN game tree and DDQN leaf-evaluation structure for Alpha–Beta Minimax and AB–DDQN Hybrid controllers.

To reduce computation, only the top-ranked candidate actions are expanded, and α – β pruning removes branches that cannot improve the final minimax decision. Terminal nodes are evaluated using a belief-aware cost based on tracking error, heading error, velocity error, control effort, and belief uncertainty.

The AB–DDQN Hybrid controller extends Alpha–Beta search by using DDQN-based leaf evaluation. The terminal value is computed using Equation 74:

$$V_{\text{leaf}}=\lambda_{\text{NN}}V_{\text{DDQN}}+\lambda_{\text{cost}}\left(-J_{\text{model}}\right),$$ (74)

where $\lambda_{\text{NN}}=0.7$ and $\lambda_{\text{cost}}=0.3$ . The DDQN value is obtained as shown in Equation 75:

$$V_{\text{DDQN}}=\underset{a\in A}{\max}Q_{\theta }\left(s,a\right).$$ (75)

Thus, the AB–DDQN Hybrid controller combines worst-case lookahead with learned long-term value estimation. This makes it a strong search-learning baseline for comparison with the proposed SSS*–DDQN controller (Ogunmolu and Gans, 2018; Wang et al., 2024b; An et al., 2026; Li et al., 2019).

## 4 Proposed SSS*–DDQN solution-space best-first search controller

This section presents the proposed SSS*–DDQN controller for robust Mecanum robot tracking under partial observability. The controller integrates EKF-based belief estimation, QMDP–DDQN action ranking, and SSS* best-first search to select reliable corrective actions (Tsai et al., 2026b; Ogunmolu and Gans, 2018; Wang et al., 2024b; An et al., 2026).

Robot action selection is modeled as a MAX decision, while disturbance response is modeled as a MIN decision. The SSS* search uses an OPEN priority queue with LIVE and SOLVED nodes to refine promising solution clusters. DDQN-based leaf evaluation is combined with model-based tracking cost to balance learned value estimation and physical tracking accuracy (Ogunmolu and Gans, 2018; Wang et al., 2024b; An et al., 2026; Li et al., 2019).

Beta-bound pruning, delayed MIN-sibling refinement, bounded OPEN-list control, and secondary lookahead improve robustness and reduce computation. Thus, the proposed SSS*–DDQN framework supports belief-aware and learning-assisted action selection under uncertainty (Jestel et al., 2026; Tsai et al., 2026a; Slaoui, 2026; Pham et al., 2025).

### 4.1 SSS* search principle and proposed control architecture

The proposed SSS*–DDQN controller is a belief-aware best-first search method for robust Mecanum robot tracking under partial observability. It integrates EKF belief estimation, QMDP–DDQN action ranking, and SSS* search refinement. At each sampling instant, the EKF belief state forms the root node, and only the top-ranked DDQN corrective actions are passed to the SSS* search to reduce computation (Tsai et al., 2026b; Ogunmolu and Gans, 2018; Wang et al., 2024b; An et al., 2026).

The SSS* search uses a three-ply MAX–MIN–MAX structure, where MAX nodes represent robot action selection and MIN nodes represent worst-case disturbance or uncertainty. Unlike depth-first Alpha–Beta search, SSS* maintains an OPEN priority queue with LIVE and SOLVED nodes to refine the most promising partial solution first. Terminal nodes are evaluated using the DDQN value blended with model-based tracking cost (Ogunmolu and Gans, 2018; Pham et al., 2025; Li et al., 2019).

As shown in Figure 4, the architecture combines reference tracking, EKF belief estimation, QMDP–DDQN action ranking, SSS* best-first search, and nominal torque compensation. Beta-bound pruning, delayed MIN-sibling refinement, bounded OPEN-list management, and secondary lookahead further improve robustness and reduce finite-horizon decision errors (Jestel et al., 2026; Tsai et al., 2026a; Slaoui, 2026; Pham et al., 2025; Yao et al., 2024; Yao et al., 2025b; Yao et al., 2025a).

**FIGURE 4:**
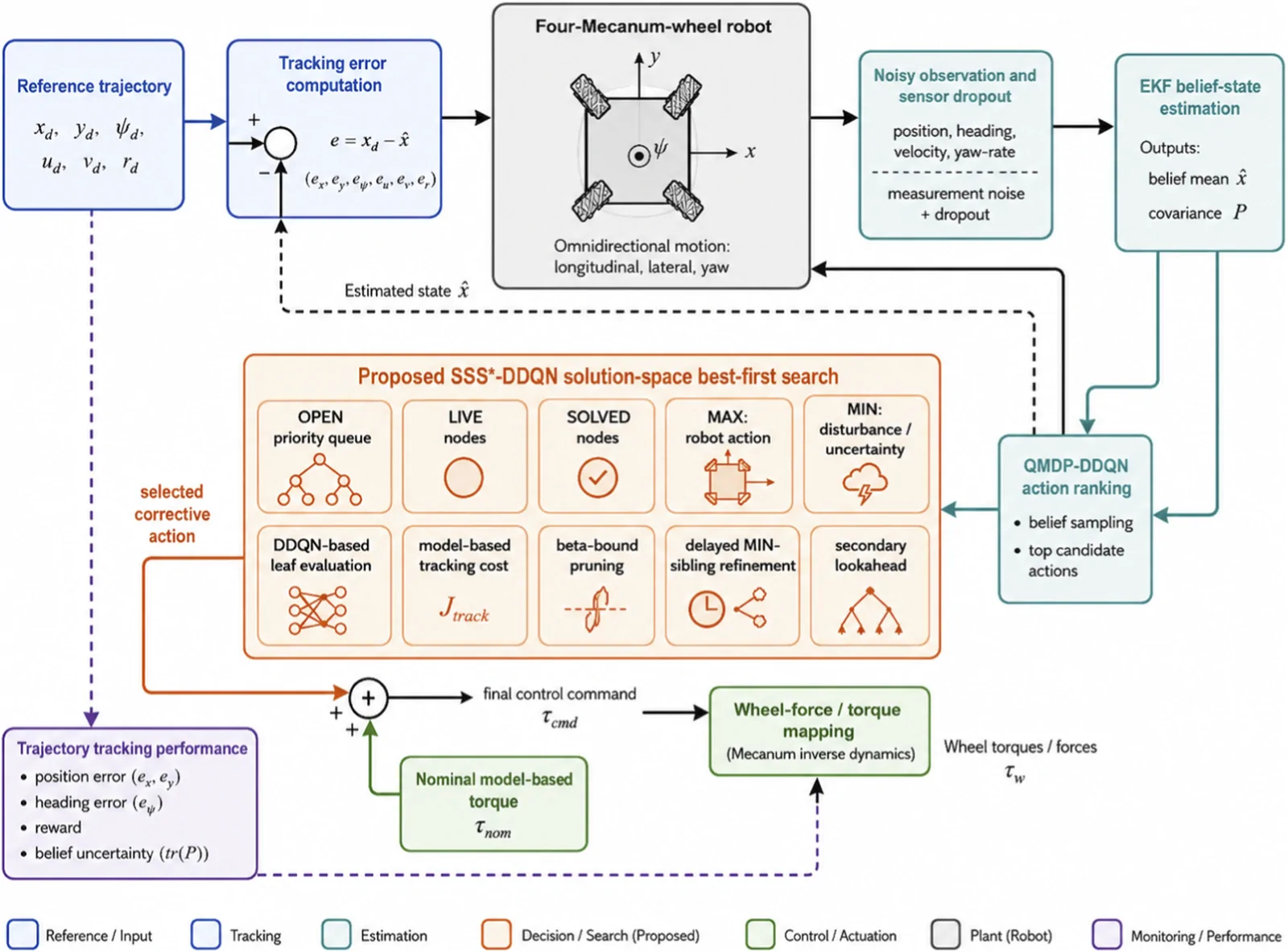
SSS*–DDQN control architecture for robust Mecanum robot trajectory tracking under partial observability.

### 4.2 LIVE/SOLVED node representation and OPEN-list refinement

The proposed SSS*–DDQN controller uses LIVE and SOLVED node labels to manage the solution-space search. Each node stores the robot state, belief covariance, search depth, player type, node status, bound value, root action, accumulated cost, and cluster identity, as shown in Equation 76:

$$N_{i}=\left(\eta_{i},\zeta_{i},P_{i},d_{i},\rho_{i},\sigma_{i},h_{i},a_{i}^{0},J_{i},c_{i}\right).$$ (76)

Here, $\rho_{i}$ denotes the player type (Equation 77), and $\sigma_{i}$ denotes the node status (Equation 78):

$$\rho_{i}\in \left\{MAX,MIN\right\},$$ (77)

$$\sigma_{i}\in \left\{LIVE,SOLVED\right\}.$$ (78)

MAX nodes represent robot corrective-action selection, while MIN nodes represent disturbance or uncertainty response. LIVE nodes require further refinement, whereas SOLVED nodes represent evaluated clusters (Ogunmolu and Gans, 2018; Wang et al., 2024b; An et al., 2026).

The OPEN list stores active nodes as Equation 79:

$$O_{k}=\left\{N_{1},N_{2},\ldots ,N_{M_{k}}\right\}.$$ (79)

At each refinement step, the highest-priority node is selected, as presented in Equation 80:

$$N^{*}=\underset{N_{i}\in O_{k}}{arg\;max}\Pi \left(N_{i}\right),$$ (80)

where the priority score is defined using Equation 81:

$$\Pi \left(N_{i}\right)=h_{i}+\lambda_{\text{s}}I\left(\sigma_{i}=SOLVED\right)+\lambda_{\text{d}}d_{i}-\lambda_{\text{l}}l_{i}.$$ (81)

When a LIVE MAX node is selected, only the top QMDP–DDQN-ranked actions are expanded, as shown in Equation 82:

$$a\in A_{\text{top}}\subset A.$$ (82)

The corresponding child state is generated as shown in Equation 83:

$$x_{i+1}=f\left(x_{i},\tau_{\text{nom}}+a\right),\;a\in A_{\text{top}}.$$ (83)

This LIVE/SOLVED and OPEN-list structure allows the controller to refine promising solution clusters instead of expanding the full game tree. As a result, the proposed SSS*–DDQN controller improves search efficiency and supports real-time robust action selection under partial observability (Ogunmolu and Gans, 2018; Jestel et al., 2026; Tsai et al., 2026a; Yu et al., 2023).

For a MIN node, the disturbance is selected from the disturbance set as shown in Equation 84:

w∈W. (84)

The disturbed state transition is shown in Equation 85:

$$x_{i+1}=f\left(x_{i},\tau_{\text{cmd}}\right)+w,\;w\in W.$$ (85)

Thus, the MIN node evaluates worst-case uncertainty before action selection.

When a LIVE node reaches the search horizon, it is converted into a SOLVED node, as shown in Equation 86:

$$LIVE\left(N_{i}\right)\to SOLVED\left(N_{i}\right),\;h_{i}=V_{\text{leaf}}\left(x_{i},P_{i},a_{i}\right).$$ (86)

The leaf value combines the DDQN value and model-based tracking cost, as presented in Equation 87:

$$V_{\text{leaf}}=\lambda_{\text{NN}}V_{\text{DDQN}}+\lambda_{\text{cost}}\left(-J_{\text{model}}\right).$$ (87)

where (Equation 88)

$$V_{\text{DDQN}}=\underset{a\in A}{\max}Q_{\theta }\left(s_{i},a\right),$$ (88)

and (Equation 89)

$$\begin{array}{cc} J_{\text{model}}= & w_{\text{p}}e_{xy}^{2}+w_{\psi }e_{\psi }^{2}+w_{\text{v}}{\left\|\zeta_{\text{d}}-\zeta \right\|}_{2}^{2}\\ & +w_{\text{u}}{\left\|a\right\|}_{2}^{2}+w_{\text{P}}\text{tr }\left(P\right). \end{array}$$ (89)

After expansion, the OPEN list is updated as Equation 90:

$$O_{k+1}=\left(O_{k}\backslash \left\{N^{*}\right\}\right)\cup C\left(N^{*}\right),$$ (90)

and bounded for real-time execution, as shown in Equation 91:

$$\left|O_{k+1}\right|\leq M_{\text{max}}.$$ (91)

If required, only the best-priority nodes are retained, as shown in Equation 92:

$$O_{k+1}={\text{Top }}_{M_{\text{max}}}\left(O_{k+1},\Pi \right).$$ (92)

Finally, the best SOLVED node determines the root action, as presented in Equation 93:

$$a_{k}^{*}=a_{i}^{0},\;i=\underset{\begin{array}{c} N_{i}\in O_{k},\\ \sigma_{i}=SOLVED \end{array}}{arg\;max}h_{i}.$$ (93)

As shown in Figure 5, the LIVE/SOLVED representation and OPEN-list refinement allow the controller to focus on promising solution clusters instead of expanding the full game tree. This improves search efficiency, robustness, and real-time suitability under partial observability (Pham et al., 2025; Beinhofer et al., 2013; Yao et al., 2024; Yao et al., 2025b; Yao et al., 2025a).

**FIGURE 5:**
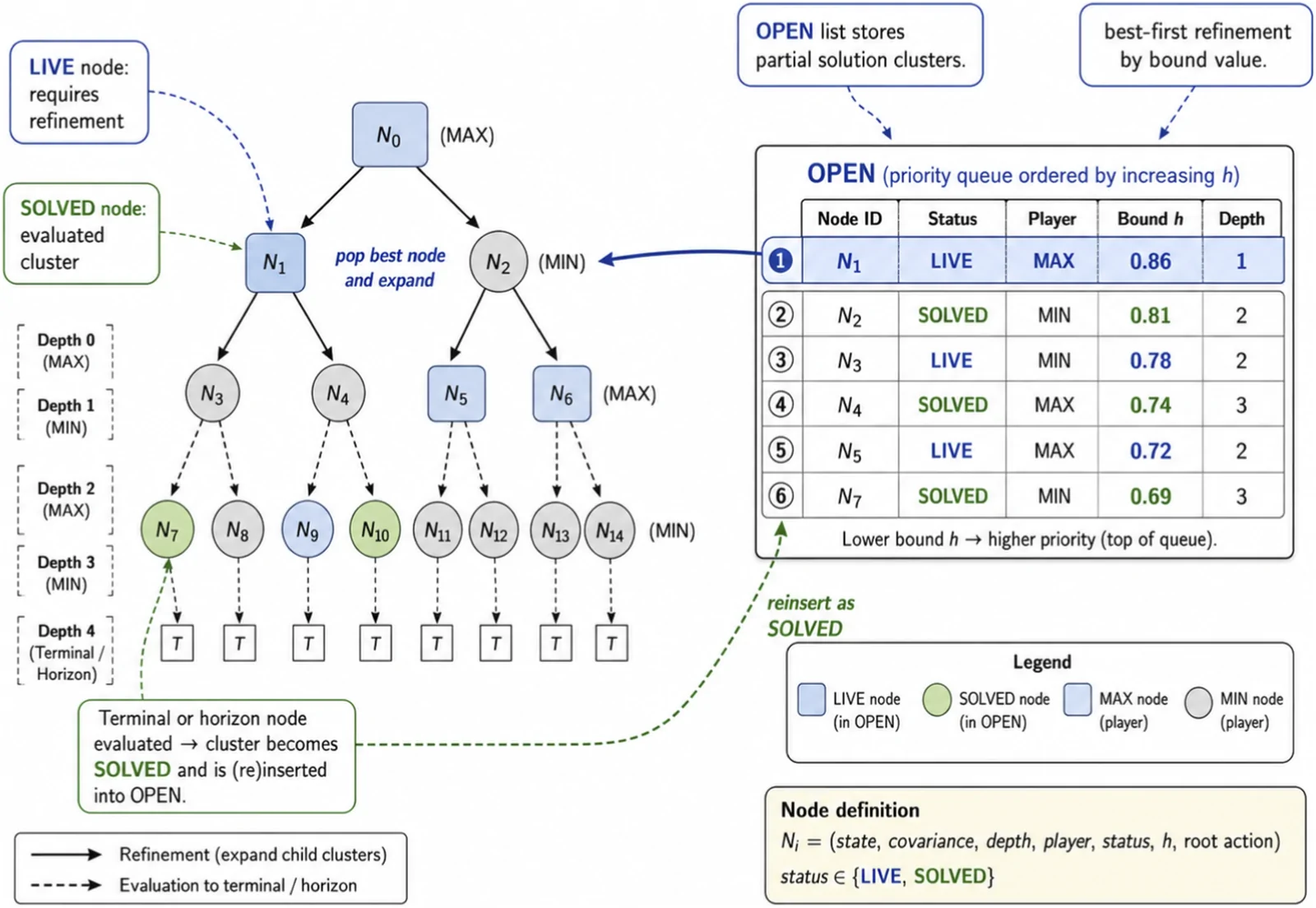
SSS* OPEN-list LIVE and SOLVED node structure used in the proposed SSS*–DDQN controller.

### 4.3 DDQN-based leaf-node evaluation with belief samples

In the proposed SSS*–DDQN controller, terminal or horizon nodes are evaluated using DDQN-assisted belief sampling. When a LIVE node reaches the search depth or reference horizon, it is converted into a SOLVED node by computing its leaf value.

For a leaf node, the estimated robot state is presented in Equation 94:

$${\hat{x}}_{i}={\left[\begin{array}{cc} \eta_{i}^{\text{T}} & \zeta_{i}^{\text{T}} \end{array}\right]}^{\text{T}},$$ (94)

where $\eta_{i}$ and $\zeta_{i}$ denote posture and body-frame velocity. The belief covariance is symmetrized as shown in Equation 95:

$$P_{i}^{\text{s}}=\frac{P_{i}+P_{i}^{\text{T}}}{2}+\epsilon I_{6},$$ (95)

to support stable belief sampling.

Belief samples are generated around the estimated state as shown in Equation 96:

$$x_{i}^{\left(j\right)}={\hat{x}}_{i}+L_{i}\xi^{\left(j\right)},\;j=1,2,\ldots ,N_{\text{b}},$$ (96)

where $L_{i}$ is the Cholesky factor of $P_{i}^{s}$ and $\xi^{\left(j\right)}$ is a Gaussian random vector.

For each sample, the DDQN input state is formed as shown in Equation 97:

$$s_{i}^{\left(j\right)}=\Phi \left(x_{i}^{\left(j\right)},\eta_{\text{d},i},{\dot{\eta }}_{\text{d},i},e_{i-1}\right),$$ (97)

and normalized using Equation 98:

$${\bar{s}}_{i}^{\left(j\right)}=\frac{s_{i}^{\left(j\right)}}{s_{\text{scale}}}.$$ (98)

The DDQN value for each sample is computed using Equation 99:

$$V_{\text{DDQN}}^{\left(j\right)}=\underset{a\in A}{\max}Q_{\theta }\left({\bar{s}}_{i}^{\left(j\right)},a\right).$$ (99)

The mean DDQN value is then obtained as Equation 100:

$${\bar{V}}_{\text{DDQN}}=\frac{1}{N_{b}}\overset{N_{b}}{\underset{j=1}{\sum }}V_{\text{DDQN}}^{\left(j\right)}.$$ (100)

Thus, belief sampling allows the leaf node to be evaluated over several possible hidden states, improving robustness under partial observability (Tsai et al., 2026b; Wang et al., 2024b; An et al., 2026; Li et al., 2019).

A model-based tracking cost is computed for each belief sample as shown in Equation 101:

$$\begin{array}{cc} J_{\text{model}}^{\left(j\right)}= & w_{\text{p}}{\left\|e_{xy}^{\left(j\right)}\right\|}_{2}^{2}+w_{\psi }{\left(e_{\psi }^{\left(j\right)}\right)}^{2}\\ & +w_{\text{v}}{\left\|\zeta_{\text{d}}^{\left(j\right)}-\zeta_{i}^{\left(j\right)}\right\|}_{2}^{2}+w_{\text{u}}{\left\|u_{i}\right\|}_{2}^{2}. \end{array}$$ (101)

The average model-based cost is shown in Equation 102:

$${\bar{J}}_{\text{model}}=\frac{1}{N_{\text{b}}}\overset{N_{\text{b}}}{\underset{j=1}{\sum }}J_{\text{model}}^{\left(j\right)}.$$ (102)

The final SSS* leaf value is obtained by combining the averaged DDQN value, negative model-based cost, and belief-uncertainty penalty, as shown in Equation 103:

$$\begin{array}{cc} V_{\text{leaf}}= & \lambda_{\text{NN}}{\bar{V}}_{\text{DDQN}}+\lambda_{\text{cost}}\left(-{\bar{J}}_{\text{model}}\right)\\ & -w_{\text{P}}\text{tr }\left(P_{i}\right). \end{array}$$ (103)

Here, $\lambda_{\text{NN}}=0.7$ and $\lambda_{\text{cost}}=0.3$ . This formulation gives higher importance to the learned DDQN value while preserving physical tracking consistency through the model-based cost. The covariance trace penalizes high-uncertainty leaf nodes (Hendzel and Kołodziej, 2021; Tsai et al., 2026b; Ogunmolu and Gans, 2018; Li et al., 2019; Li et al., 2024).

Thus, the leaf-node evaluation combines learned long-term value, model-based tracking accuracy, and belief uncertainty. Algorithm 3 summarizes the DDQN-assisted SSS* leaf-node evaluation procedure used in the proposed controller (Yao et al., 2024; Yao et al., 2025b; Yao et al., 2025a; Li et al., 2019; Li et al., 2024).

Algorithm 3 DDQN-assisted SSS* leaf-node evaluation.

1. Input: Leaf node state, belief covariance, trained DDQN network, action set, reference trajectory, previous tracking error, and state-scaling vector.
2. Extract the estimated robot posture, body-frame velocity, and belief covariance from the selected SSS* leaf node.
3. Symmetrize the belief covariance matrix to ensure numerical stability.
4. Generate belief samples around the EKF-estimated leaf state using the covariance matrix.
5. For each belief sample,
  - Extract sampled position, heading, velocity, and yaw-rate information.
  - Construct the DDQN input state using sampled belief state, tracking error, reference velocity, and reference posture.
  - Normalize the DDQN input state using the state-scaling vector.
  - Evaluate Q-values for all discrete corrective actions using the trained DDQN network.
  - Select the maximum Q-value as the DDQN value of the sampled state.
  - Compute the model-based tracking cost using position error, heading error, velocity error, control effort, and belief uncertainty.
6. Average the DDQN values over all belief samples.
7. Average the model-based tracking costs over all belief samples.
8. Compute the final leaf value by blending the averaged DDQN value and the negative model-based tracking cost.
9. Add the belief-uncertainty penalty using the covariance trace.
10. Convert the LIVE leaf node into a SOLVED node using the computed leaf value.
11. Reinsert the SOLVED node into the SSS* OPEN priority queue.
12. Output: SOLVED leaf node and DDQN-assisted leaf value.

### 4.4 Beta-bound pruning, delayed MIN-sibling refinement, and secondary lookahead

The proposed SSS*–DDQN controller uses beta-bound pruning, delayed MIN-sibling refinement, and secondary lookahead to improve robustness and reduce computation. In SSS* search, each node has a bound value h , which represents the current quality estimate of a partial solution cluster (Ogunmolu and Gans, 2018; Jestel et al., 2026; Pham et al., 2025).

When a LIVE node reaches the search horizon, the raw leaf value is computed as shown in Equation 104:

$$V_{\text{raw}}=V_{\text{leaf}}\left(N_{i}\right).$$ (104)

The beta-bound rule limits this value using the inherited bound, as shown in Equation 105:

$$h_{i}^{\text{new}}=\min\left(h_{i},V_{\text{raw}}\right).$$ (105)

If the raw value exceeds the inherited bound, it is not allowed to improve the parent cluster, as shown in Equation 106:

$$V_{\text{raw}}>h_{i}\;\Rightarrow \;h_{i}^{\text{new}}=h_{i}.$$ (106)

This prevents over-optimistic DDQN leaf estimates from dominating the final decision and supports safer action selection under partial observability (Tsai et al., 2026b; Ogunmolu and Gans, 2018; Yao et al., 2024; Yao et al., 2025b; yao et al., 2025a).

At MIN nodes, disturbance branches are ranked using the tracking-error danger score, as presented in Equation 107:

$$D_{j}=w_{\text{p}}{\left\|e_{xy,j}\right\|}_{2}^{2}+w_{\psi }e_{\psi ,j}^{2}.$$ (107)

The most critical disturbance is refined first, as shown in Equation 108:

$$w^{*}=\underset{w_{j}\in W}{arg\;max}D_{j}.$$ (108)

Less critical MIN siblings are delayed in the OPEN list with reduced priority, as shown in Equation 109:

$$h_{j}^{\text{delayed}}=h_{i}-\delta_{\text{MIN}}j,\;j>1.$$ (109)

This reduces unnecessary disturbance expansion while preserving important worst-case branches (Ogunmolu and Gans, 2018; Pham et al., 2025; Beinhofer et al., 2013).

To control computation, the OPEN list is bounded by retaining only the highest-priority nodes, as shown in Equation 110:

$$O_{k+1}={\text{Top }}_{M_{\text{max}}}\left(O_{k+1}\right).$$ (110)

A secondary lookahead is then applied to reduce finite-horizon errors. For each candidate action, the secondary score is computed as shown in Equation 111:

$$S\left(a_{i}\right)=V_{\text{leaf}}\left(a_{i}\right)-\lambda_{\text{h}}\overset{d_{\text{s}}}{\underset{q=1}{\sum }}D_{q}\left(a_{i}\right).$$ (111)

The final action is selected as shown in Equation 112:

$$a_{k}^{*}=\underset{a_{i}\in A_{\text{s}}}{arg\;max}S\left(a_{i}\right).$$ (112)

Thus, delayed MIN-sibling refinement, bounded OPEN-list management, and secondary lookahead improve robustness and computational efficiency (Mehta et al., 2025; Jaramillo-Martínez and Chavero-Navarrete, 2026; Yu et al., 2023; Li et al., 2019; Li et al., 2024; Xu et al., 2026; Lluvia Hermosilla, 2023; Zhou et al., 2019; Liao et al., 2025). Algorithm 4 summarizes the complete online SSS*–DDQN action-selection process, including top-ranked DDQN action expansion, OPEN-list refinement, LIVE/SOLVED node handling, beta-bound pruning, delayed MIN-sibling refinement, secondary lookahead, and final control-command generation.

Algorithm 4 Complete SSS*–DDQN online action-selection logic.

1. Input: EKF belief state, belief covariance, reference trajectory, trained DDQN network, action set, disturbance set, SSS* search parameters, and robot model parameters.
2. Output: Robust corrective action and final control command.
3. Initialize the current robot belief mean and covariance from the EKF.
4. Compute the tracking error using the reference trajectory and the EKF-estimated robot state.
5. Construct the DDQN input state from the EKF belief state, tracking error, previous error, and reference information.
6. Normalize the DDQN input state using the state-scaling vector.
7. Evaluate Q-values for all discrete corrective actions using the trained DDQN network.
8. Rank the candidate actions based on QMDP–DDQN action values.
9. Select the top-ranked candidate actions for SSS* search.
10. Create the root SSS* node using the current belief state.
11. Insert the root node into the OPEN priority queue as a LIVE node.
12. While the OPEN list is not empty and the maximum search iteration is not reached,
  - Select the highest-priority node from the OPEN list.
  - If the selected node reaches the search horizon,
    (1) Generate belief samples around the node state using the EKF covariance.
    (2) Evaluate the node using the trained DDQN network.
    (3) Compute the model-based tracking cost.
    (4) Blend the DDQN value and model-based cost.
    (5) Apply the belief-uncertainty penalty.
    (6) Convert the LIVE node into a SOLVED node.
    (7) Reinsert the SOLVED node into the OPEN list.
  - Else if the selected node is a MAX node,
    (1) Expand the top-ranked robot corrective actions.
    (2) Simulate each child state using the Mecanum robot model.
    (3) Assign each child node as a MIN node.
    (4) Insert the generated child nodes into the OPEN list.
  - Else if the selected node is a MIN node,
    (1) Evaluate disturbance branches using tracking-error severity.
    (2) Select the most dangerous disturbance branch for immediate refinement.
    (3) Delay the remaining MIN-sibling branches using reduced priority.
    (4) Insert the refined and delayed child nodes into the OPEN list.
  - Apply beta-bound pruning to prevent over-optimistic leaf values.
  - If the OPEN list exceeds its maximum size, retain only the highest-priority nodes.
13. Select the best SOLVED node from the OPEN list.
14. Extract the corresponding root corrective action.
15. Apply secondary lookahead to check for hidden finite-horizon disturbance effects.
16. If secondary lookahead finds a safer action, replace the selected action.
17. Compute the nominal model-based torque command.
18. Combine the nominal torque and the selected SSS*–DDQN corrective action.
19. Apply the final control command to the Mecanum robot.
20. Update the EKF belief state using the next noisy observation.
21. Repeat the procedure at the next sampling instant.

### 4.5 Formal integration of DDQN value estimation with SSS* search

The proposed SSS*–DDQN method integrates DDQN value prediction with SSS* search at four levels: action ranking, node expansion, leaf-node evaluation, and final action selection. The EKF prediction–correction mechanism also provides an explicit belief-consistency operation. The nonlinear robot model predicts the next state, while the innovation term and Kalman gain correct this prediction using the latest noisy observation. This role is conceptually related to state-consistency regularization in learned latent-dynamics methods, but the proposed framework enforces consistency through probabilistic filtering rather than through an auxiliary self-supervised loss. Therefore, DDQN and SSS* operate on an observation-corrected belief state instead of an unconstrained latent state.

At each control step, the current belief-based state $s_{k}$ is evaluated using the trained DDQN network as shown in Equation 113:

$$Q_{k}\left(a\right)=Q_{\theta }\left(s_{k},a\right),\;a\in A.$$ (113)

The DDQN values are used to rank all candidate corrective actions. Only the top-ranked actions are selected for SSS* expansion, as shown in Equation 114:

$$A_{\text{top}}={\text{Top }}_{K}\left(A,Q_{\theta }\left(s_{k},a\right)\right).$$ (114)

Thus, DDQN reduces the branching factor by allowing the SSS* search to refine only the most promising action candidates. For a LIVE MAX node, child nodes are generated using Equation 115:

$$x_{i+1}=f\left(x_{i},\tau_{\text{nom}}+a_{i}\right),\;a_{i}\in A_{\text{top}}.$$ (115)

At a terminal or horizon node, DDQN is used for leaf-value estimation. The DDQN value is computed as shown in Equation 116:

$$V_{\text{DDQN}}=\underset{a\in A}{\max}Q_{\theta }\left(s_{i},a\right).$$ (116)

The final leaf value combines learned DDQN value, model-based tracking cost, and belief uncertainty, as shown in Equation 117:

$$V_{\text{leaf}}=\lambda_{\text{NN}}V_{\text{DDQN}}+\lambda_{\text{cost}}\left(-J_{\text{model}}\right)-w_{\text{P}}\text{tr }\left(P_{i}\right).$$ (117)

This value is used to convert LIVE nodes into SOLVED nodes, as shown in Equation 118:

$$LIVE\left(N_{i}\right)\to SOLVED\left(N_{i}\right),\;h_{i}=V_{\text{leaf}}.$$ (118)

The beta-bound update is then applied as shown in Equation 119:

$$h_{i}^{\text{new}}=\min\left(h_{i},V_{\text{leaf}}\right).$$ (119)

Therefore, over-optimistic DDQN leaf values are limited by the inherited SSS* bound. Finally, the selected root action is obtained from the best SOLVED node, as presented in Equation 120:

$$a_{k}^{*}=a_{i}^{0},\;i=\underset{\begin{array}{c} N_{i}\in O,\\ \sigma_{i}=SOLVED \end{array}}{arg\;max}h_{i}.$$ (120)

This formulation shows that DDQN does not independently replace the search process. Instead, DDQN guides the SSS* search by ranking candidate actions, evaluating terminal nodes, influencing pruning bounds, and supporting final robust action selection.

## 5 Results and comparative performance analysis

This section presents the simulation results of the proposed SSS*–DDQN controller for lemniscate trajectory tracking under partial observability. The performance is compared with four baseline controllers: EKF-belief PER-DDQN, QMDP-PER-DDQN, Alpha–Beta Minimax, and AB–DDQN Hybrid. All controllers are evaluated using the same Mecanum robot model, noisy observation condition, EKF belief estimation, and reference trajectory. The analysis includes trajectory tracking performance, position error, heading error, belief uncertainty, reward response, control effort, search efficiency, and overall controller ranking. This comparison highlights the ability of the proposed SSS*–DDQN method to improve robust action selection by combining belief-state estimation, learned DDQN value evaluation, and solution-space best-first search.

Figure 6 compares the desired and actual lemniscate trajectory responses of the five controllers. In Figure 6a, the EKF-belief PER-DDQN obtains ${\text{RMSE}}_{xy}$ = 0.03013 m and ${\text{MAE}}_{xy}$ = 0.02689 m. In Figure 6b, the QMDP-PER-DDQN yields ${\text{RMSE}}_{xy}$ = 0.02677 m and ${\text{MAE}}_{xy}$ = 0.02171 m. In Figure 6c, the Alpha–Beta Minimax controller achieves ${\text{RMSE}}_{xy}$ = 0.03082 m and ${\text{MAE}}_{xy}$ = 0.02965 m. In Figure 6d, the AB–DDQN Hybrid achieves the best tracking accuracy, with ${\text{RMSE}}_{xy}$ = 0.01837 m and ${\text{MAE}}_{xy}$ = 0.01536 m. In Figure 6e, the proposed SSS*–DDQN also shows strong tracking performance, with ${\text{RMSE}}_{xy}$ = 0.01955 m and ${\text{MAE}}_{xy}$ = 0.01658 m. Finally, Figure 6f presents the overall comparison, confirming that the search-enhanced DDQN-based controllers provide improved trajectory tracking under partial observability.

**FIGURE 6:**
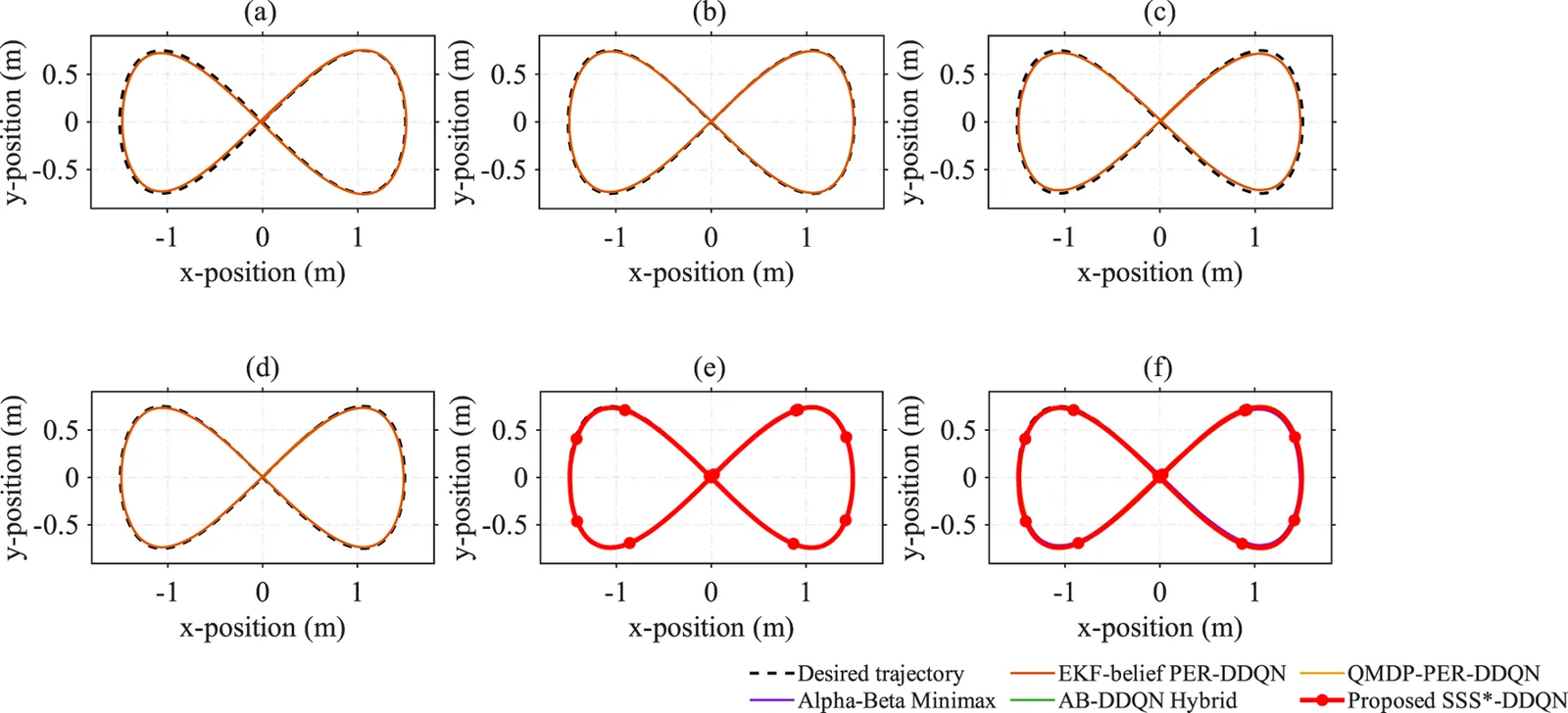
Desired and actual lemniscate trajectory comparison of five controllers. **(a)** EKF-belief, **(b)** QMDP, **(c)** Alpha-Beta, **(d)** AB-DDQN, **(e)** Proposed SSS*-DDQN, **(f)** Overall comparison.

Figure 7 shows the position tracking error responses of the five controllers. In Figure 7a, the EKF-belief PER-DDQN yields ${\text{RMSE}}_{x}$ = 0.01953 m and ${\text{RMSE}}_{y}$ = 0.02294 m. In Figure 7b, the QMDP-PER-DDQN yields ${\text{RMSE}}_{x}$ = 0.01951 m and ${\text{RMSE}}_{y}$ = 0.01833 m. In Figure 7c, the Alpha–Beta Minimax controller achieves ${\text{RMSE}}_{x}$ = 0.02049 m and ${\text{RMSE}}_{y}$ = 0.02302 m. In Figure 7d, the AB–DDQN Hybrid achieves the lowest position error, with ${\text{RMSE}}_{x}$ = 0.01204 m and ${\text{RMSE}}_{y}$ = 0.01387 m. In Figure 7e, the proposed SSS*–DDQN yields ${\text{RMSE}}_{x}$ = 0.01433 m and ${\text{RMSE}}_{y}$ = 0.01329 m. Figure 7f compares the X-error and Y-error responses of all controllers. From the results, the AB–DDQN Hybrid provides the minimum position error, while the proposed SSS*–DDQN maintains a closely competitive response with balanced X- and Y-direction tracking accuracy. Therefore, the search-enhanced DDQN controllers show improved position tracking capability under partial observability compared with the EKF-belief, QMDP, and Alpha–Beta baseline methods.

**FIGURE 7:**
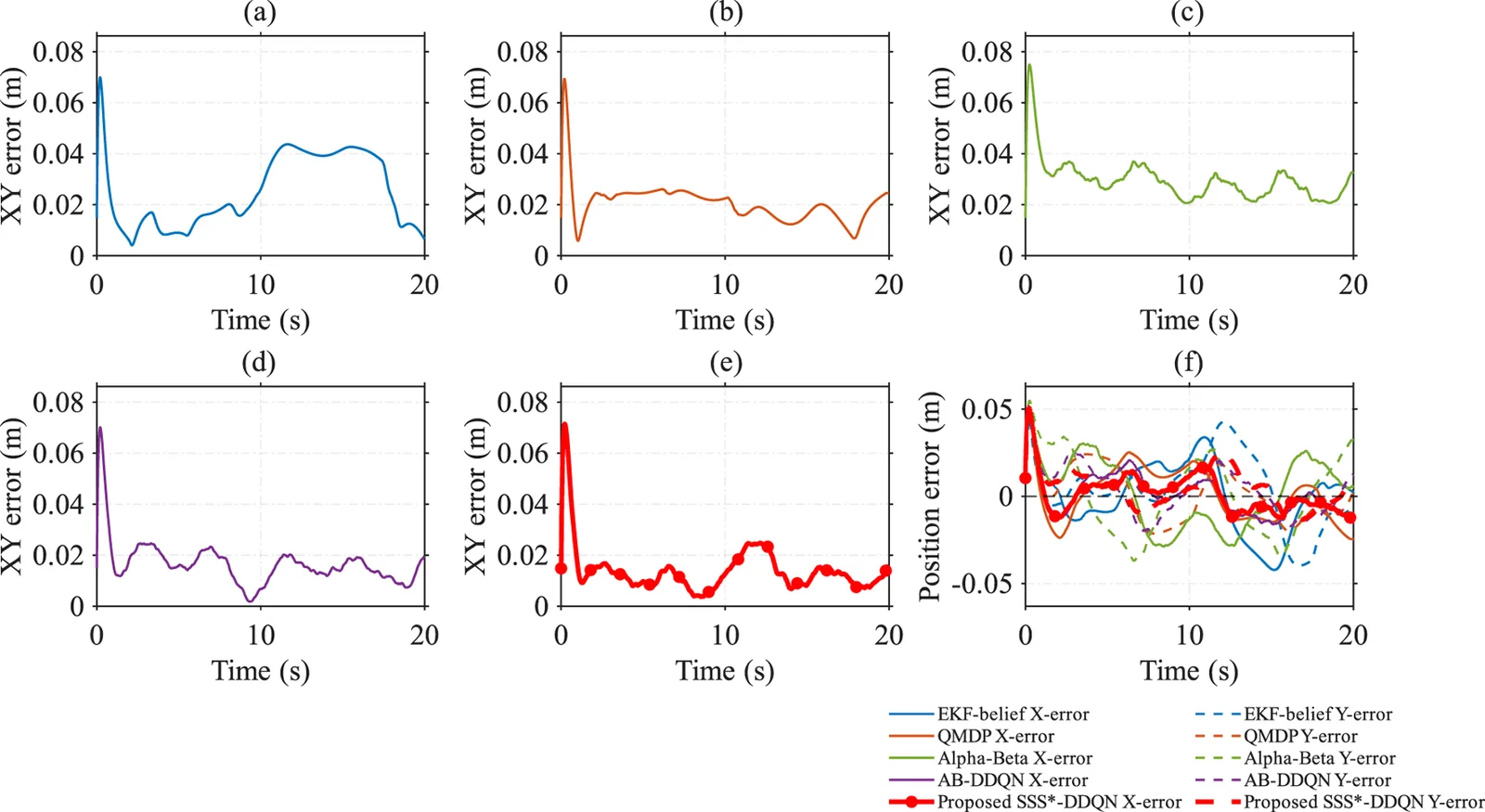
Position tracking error comparison of five controllers. **(a)** EKF-belief, **(b)** QMDP, **(c)** Alpha-Beta, **(d)** AB-DDQN, **(e)** Proposed SSS*-DDQN, **(f)** X-error and Y-error.

Figure 8 presents the heading error response of the five controllers. In Figure 8a, the EKF-belief PER-DDQN yields ${\text{RMSE}}_{\psi }$ = 0.00984 rad and ${\text{MAE}}_{\psi }$ = 0.00641 rad. In Figure 8b, the QMDP-PER-DDQN yields ${\text{RMSE}}_{\psi }$ = 0.01618 rad and ${\text{MAE}}_{\psi }$ = 0.01588 rad. In Figure 8c, the Alpha–Beta Minimax controller achieves the lowest heading error, with ${\text{RMSE}}_{\psi }$ = 0.00389 rad and ${\text{MAE}}_{\psi }$ = 0.00319 rad. In Figure 8d, the AB–DDQN Hybrid yields ${\text{RMSE}}_{\psi }$ = 0.00945 rad and ${\text{MAE}}_{\psi }$ = 0.00790 rad. In Figure 8e, the proposed SSS*–DDQN achieves ${\text{RMSE}}_{\psi }$ = 0.01288 rad and ${\text{MAE}}_{\psi }$ = 0.01147 rad Figure 8f shows the overall heading error comparison of all controllers. From the results, the Alpha–Beta Minimax controller provides the best heading accuracy, while the AB–DDQN Hybrid and EKF-belief PER-DDQN maintain moderate heading errors. The proposed SSS*–DDQN shows acceptable orientation tracking, but its main advantage is observed in position tracking accuracy rather than heading error minimization.

**FIGURE 8:**
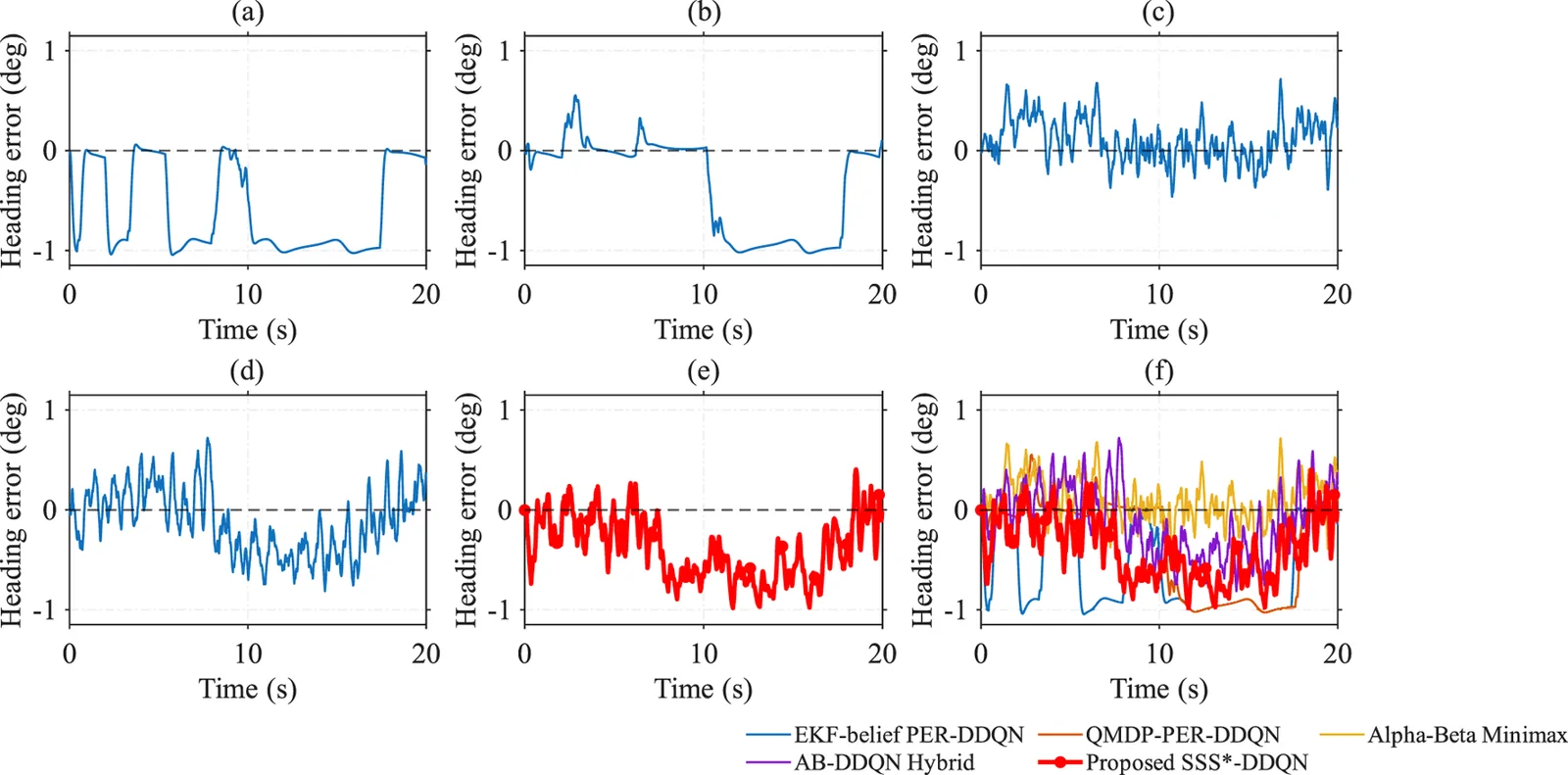
Heading error response comparison of five controllers. **(a)** EKF-belief, **(b)** QMDP, **(c)** Alpha-Beta, **(d)** AB-DDQN **(e)** Proposed SSS*-DDQN **(f)** Overall comparison.

Figure 9 compares the corrected velocity, acceleration, and torque profiles of the five controllers. In Figure 9a, all controllers show similar RMS speed values ranging from 0.52303 to 0.52906 m/s. In Figure 9b, the RMS yaw-rate values are nearly identical, ranging from 0.59866 to 0.60059 rad/s. In Figure 9c, the RMS translational acceleration varies from 0.34897 to 0.36093 ${m/s}^{2}$ . In Figure 9D, the corrected RMS yaw-acceleration remains within 0.46367–0.47541 ${rad/s}^{2}$ . In Figure 9e, the RMS wheel torque ranges from 0.18098 to 0.22457 Nm, with the same maximum torque of 1.80000 Nm for all methods. In Figure 9f, the RMS yaw torque ranges from 0.38453 to 0.41888 Nm. Overall, all controllers operate under comparable motion conditions. The proposed SSS*–DDQN maintains moderate acceleration and torque demand, and it requires lower RMS wheel torque and yaw torque than AB–DDQN Hybrid, indicating a better balance between tracking accuracy and control effort.

**FIGURE 9:**
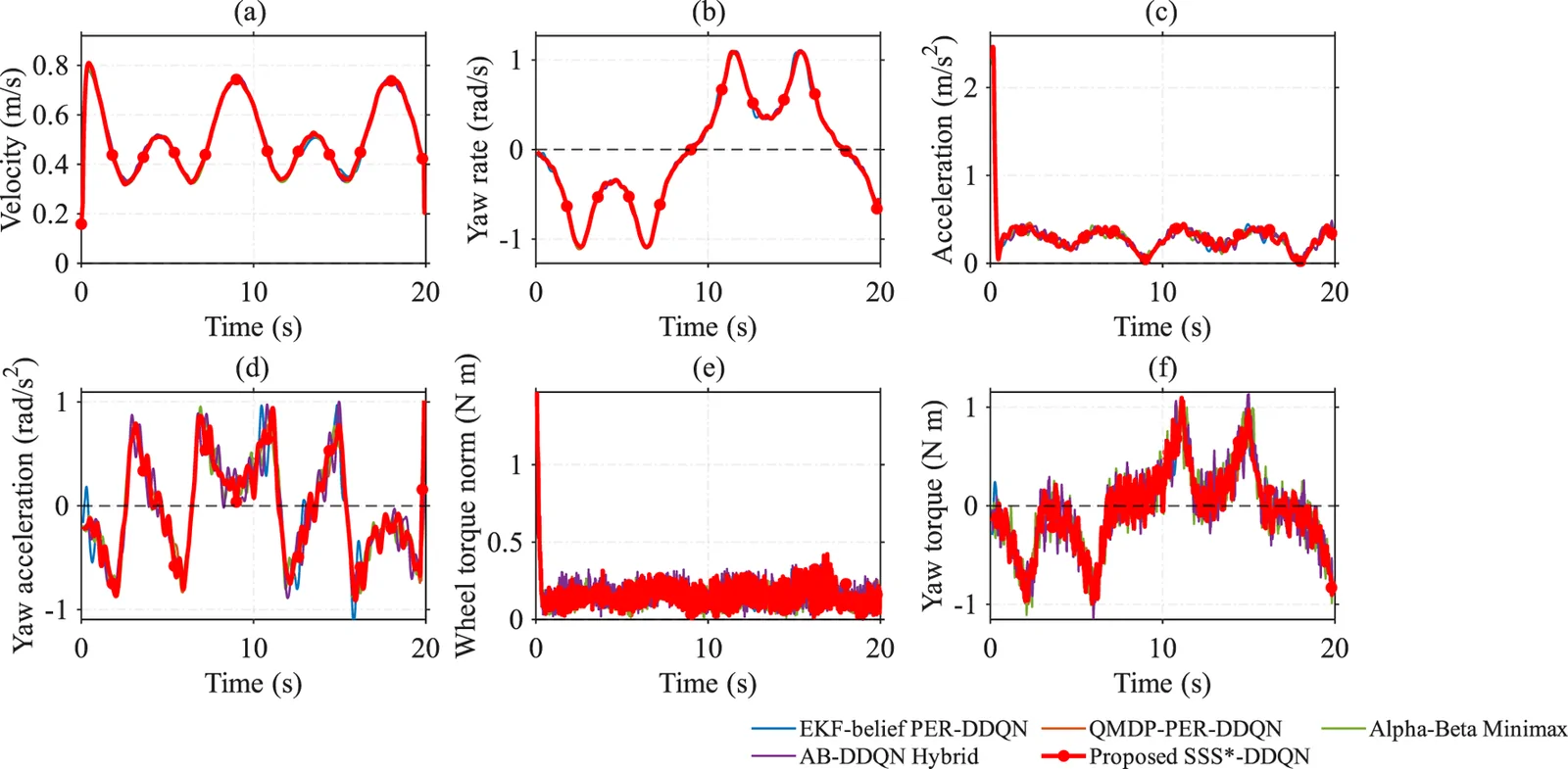
Corrected velocity, acceleration, and torque profile comparison of five controllers. **(a)** Translational velocity, **(b)** Yaw-rate profile, **(c)** Translational acceleration, **(d)** Yaw-acceleration profile, **(e)** Wheel torque profile, **(f)** Yaw torque profile.

Figure 10 shows the EKF covariance trace and belief-state convergence of the five controllers. For all methods, tr(P) decreases from 0.00533863 to approximately 0.00271338, with a mean value of approximately 0.00271803. The EKF-belief PER-DDQN, QMDP-PER-DDQN, Alpha–Beta Minimax, AB–DDQN Hybrid, and proposed SSS*–DDQN exhibit almost identical covariance convergence. This confirms stable belief-state estimation under partial observability. Hence, the performance differences among the controllers are mainly due to their action-selection and search strategies rather than estimation uncertainty.

**FIGURE 10:**
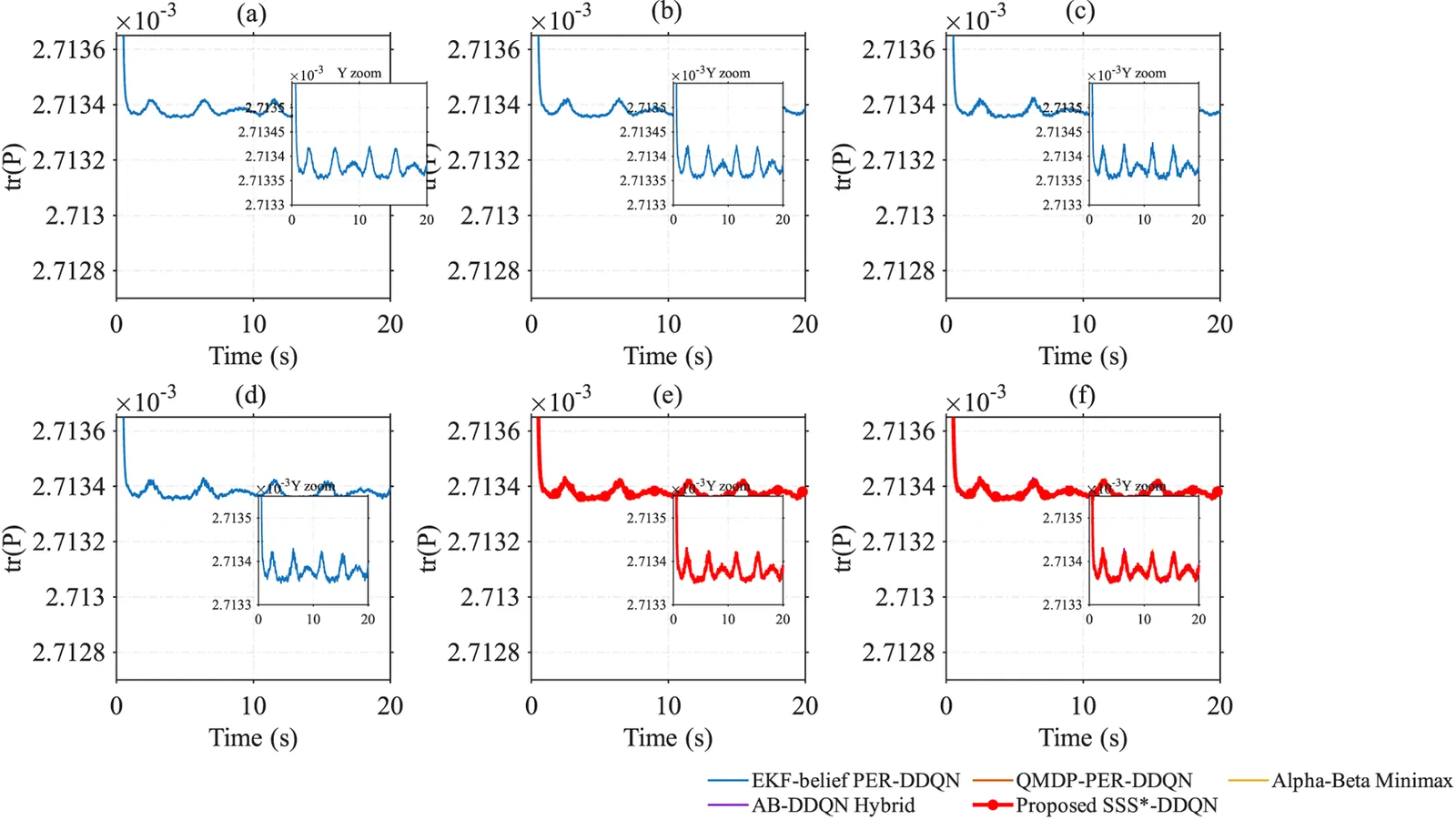
EKF covariance trace and belief-state convergence comparison of five controllers with zoomed steady-state view. **(a)** EKF-belief, **(b)** QMDP, **(c)** Alpha-Beta, **(d)** AB-DDQN, **(e)** Proposed SSS*-DDQN, **(f)** Overall comparison.

Figure 11 presents the cumulative reward and control-effort energy comparison of the five controllers.

**FIGURE 11:**
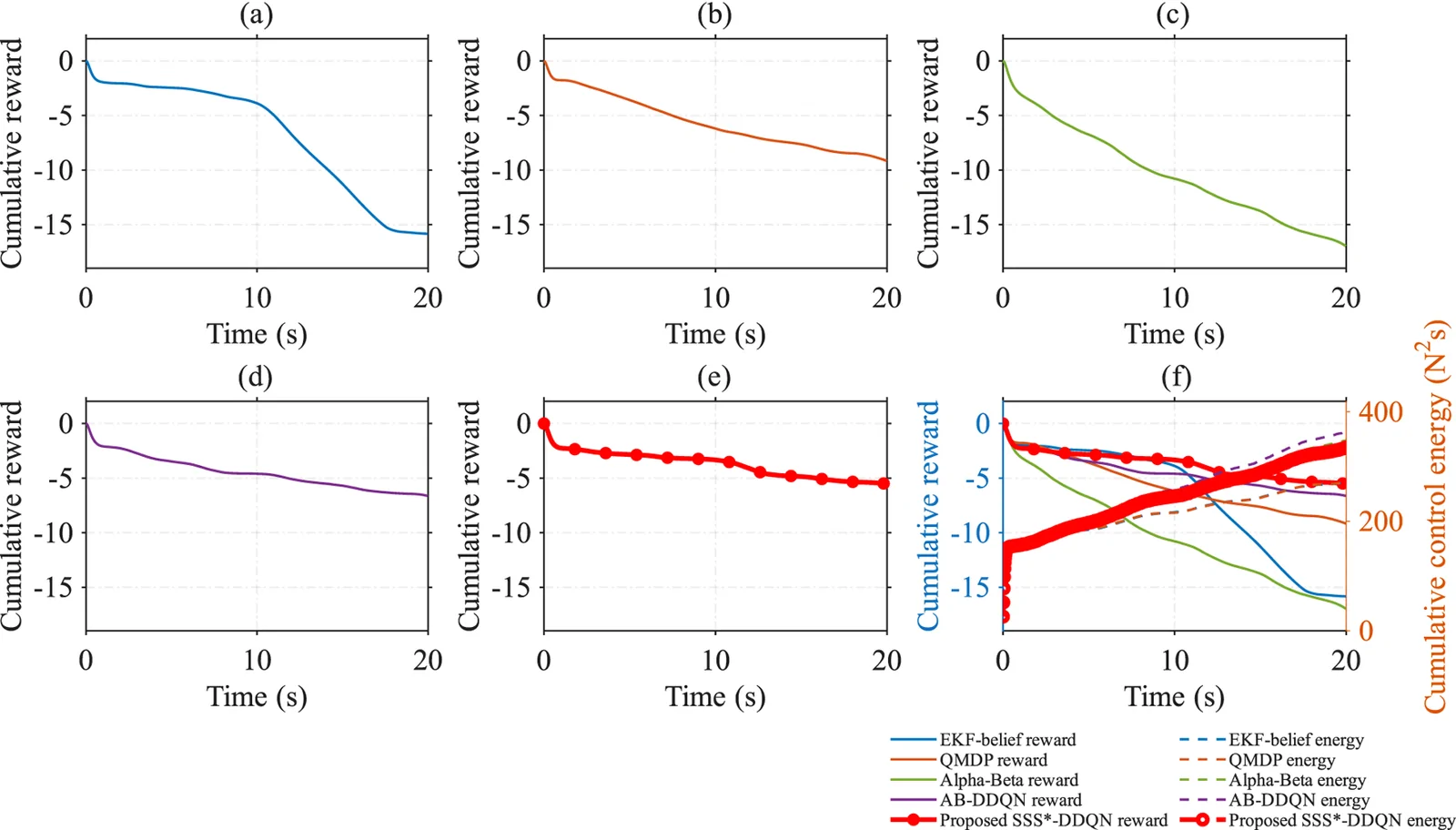
Cumulative reward and control-effort energy comparison of five controllers. **(a)** EKF-belief, **(b)** QMDP, **(c)** Alpha-Beta, **(d)** AB-DDQN, **(e)** Proposed SSS*-DDQN, **(f)** Reward and control energy.

In Figure 11a, the EKF-belief PER-DDQN achieves a final cumulative reward of −16.6775, with the total control energy of 268.4841 ${\text{N}}^{2}$ s. In Figure 11b, the QMDP-PER-DDQN yields a final cumulative reward of −13.8202, with the lowest control energy of 255.0229 ${\text{N}}^{2}$ s. In Figure 11c, the Alpha–Beta Minimax controller yields the lowest cumulative reward of −17.1499, with the control energy of 348.1435 ${\text{N}}^{2}$ s. In Figure 11d, the AB–DDQN Hybrid achieves the best cumulative reward of −6.3876, but with the highest control energy of 386.7046 ${\text{N}}^{2}$ s. In Figure 11e, the proposed SSS*–DDQN achieves a competitive cumulative reward of −7.4583, with a lower control energy of 358.0317 ${\text{N}}^{2}$ s than the AB–DDQN Hybrid. Figure 11f shows the overall reward and energy trade-off. From the results, AB–DDQN Hybrid provides the best reward performance, while QMDP-PER-DDQN requires the least control energy. The proposed SSS*–DDQN offers a balanced trade-off by maintaining near-best reward performance with lower control effort than the AB–DDQN Hybrid, indicating effective reward–energy balancing under partial observability.

Figure 12 illustrates the search-efficiency and computational performance of the five controllers using the values reported in Table 4. In Figure 12a, the EKF-belief PER-DDQN and QMDP-PER-DDQN do not refine search nodes, while Alpha–Beta Minimax, AB–DDQN Hybrid, and proposed SSS*–DDQN refine $2.642\times 10^{5}$ , $2.6039\times 10^{5}$ , and $3.9494\times 10^{5}$ nodes, respectively. In Figure 12b, only the proposed SSS*–DDQN generates additional search nodes, reaching $7.9705\times 10^{5}$ nodes. In Figure 12c, the proposed method prunes $2.5382\times 10^{5}$ nodes, which is much higher than Alpha–Beta Minimax and AB–DDQN Hybrid. In Figure 12d, the proposed SSS*–DDQN achieves the highest pruning ratio of 64.27% , compared with 13.44% and 16.06% for Alpha–Beta Minimax and AB–DDQN Hybrid, respectively. In Figure 12e, the proposed method performs 36,383 secondary lookahead operations, while the other methods do not use this mechanism. In Figure 12f, the proposed SSS*–DDQN records the highest execution index of $1.4822\times 10^{6}$ due to solution-space best-first refinement.

**FIGURE 12:**
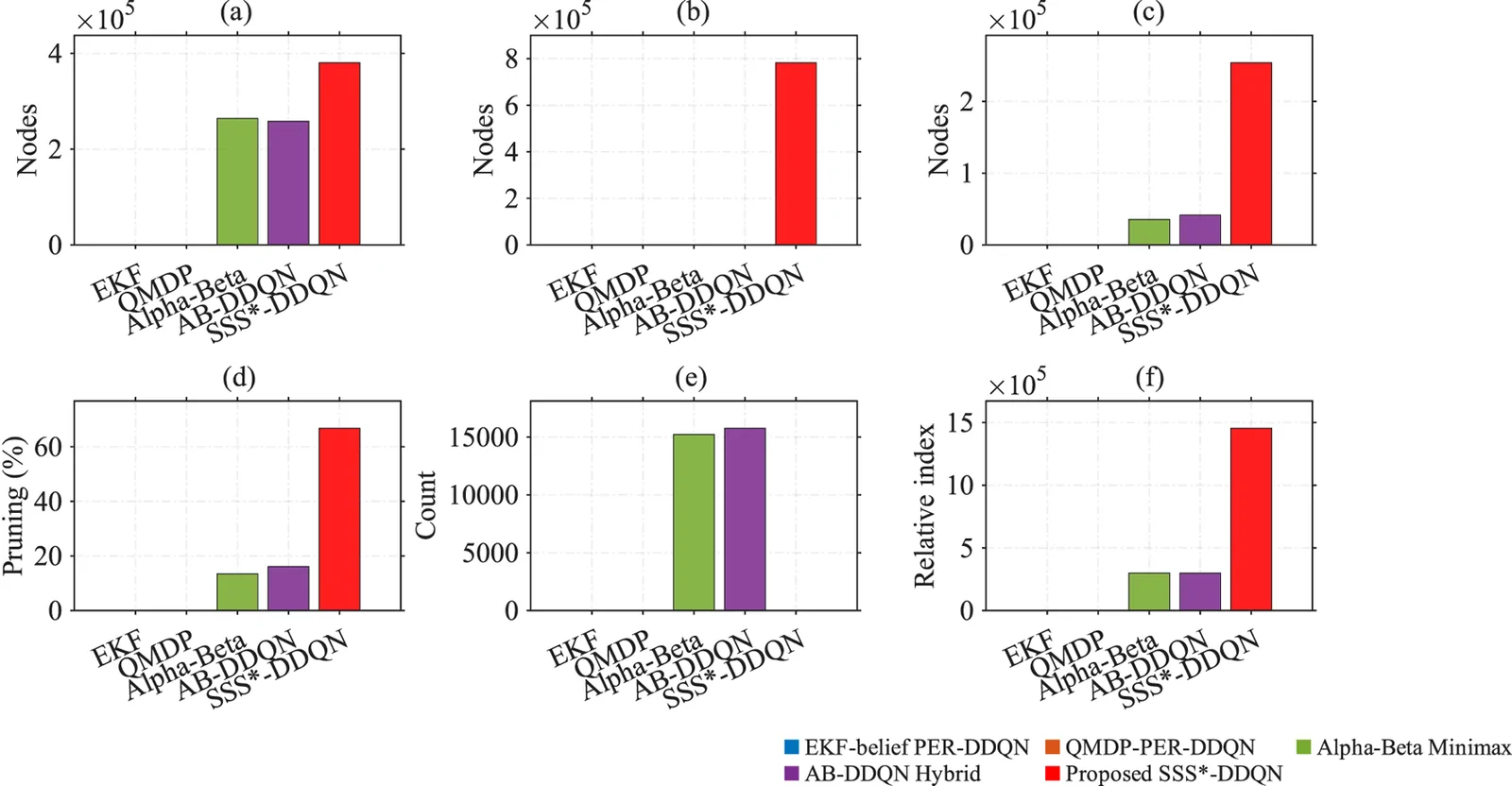
Search-efficiency and computational performance comparison. **(a)** Nodes searched/refined, **(b)** Nodes generated, **(c)** Nodes pruned, **(d)** Pruning ratio, **(e)** Cutoff / beta-bound events, **(f)** Execution-statistics index.

**TABLE 4:** Search-efficiency and computational performance of the five controllers. The best pruning ratio is highlighted in bold.

Controller	Nodes searched/Refined	Nodes generated	Nodes pruned	Pruning (%)	Secondary lookahead	Execution index
EKF-belief PER-DDQN	0	0	0	0	0	0
QMDP-PER-DDQN	0	0	0	0	0	0
Alpha–Beta Minimax	$2.642\times 10^{5}$	0	35,508	13.44	0	$2.9971\times 10^{5}$
AB–DDQN Hybrid	$2.6039\times 10^{5}$	0	41,813	16.06	0	$3.0220\times 10^{5}$
Proposed SSS*–DDQN	$3.9494\times 10^{5}$	$7.9705\times 10^{5}$	$2.5382\times 10^{5}$	**64.27**	36,383	$1.4822\times 10^{6}$

Overall, the proposed SSS*–DDQN requires higher computational effort than the baseline controllers. However, this additional cost is justified by its stronger pruning capability, secondary lookahead, and uncertainty-aware search refinement under partial observability.

Figure 13 presents the DDQN training convergence and learning stability over 70 episodes. In Figure 13a, the cumulative reward starts from 1784.18, reaches the best value of 1810.22, and gives a final smoothed reward of 1730.40. In Figure 13b, the TD loss increases from 0.56562 to 2.76598, with a minimum value of 0.44725 and mean value of 1.9765, indicating continuous value-function update during learning. In Figure 13c, the exploration rate decreases from 1.0000 to 0.57452, while the ${\text{RMSE}}_{xy}$ changes from 0.01479 m to 0.03757 m, with the best ${\text{RMSE}}_{xy}$ of 0.01282 m. Overall, the training curves show that the DDQN agent explores actively in the initial episodes and gradually shifts toward exploitation as the exploration rate decreases. The reward and RMSE variations indicate stochastic learning behaviour under partial observability, while the smoothed curves confirm stable learning progression.

**FIGURE 13:**
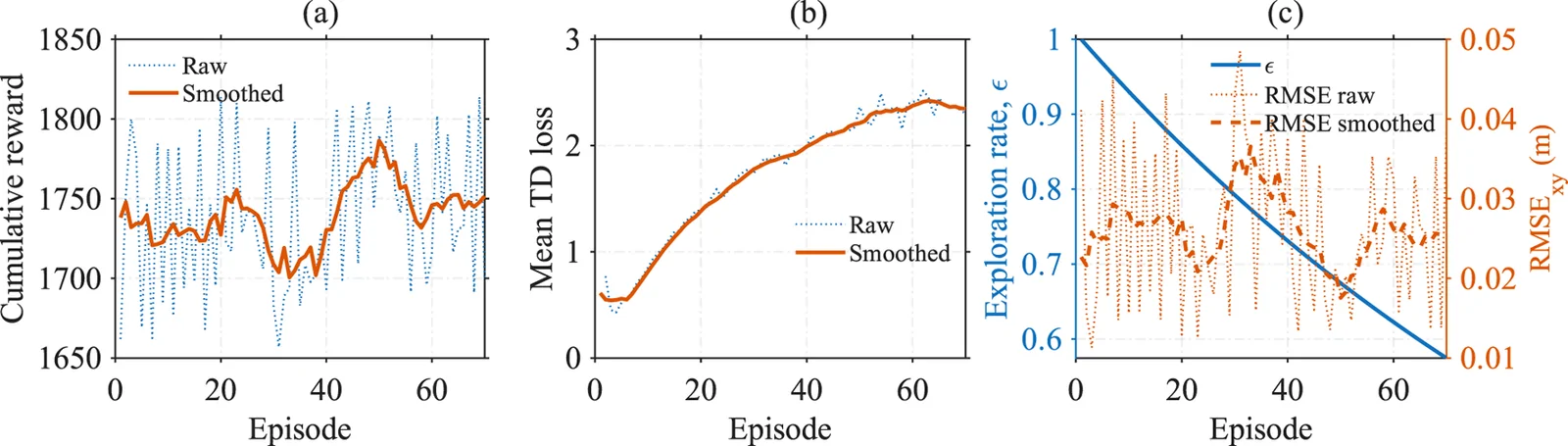
DDQN training convergence and learning stability. **(a)** Cumulative reward, **(b)** TD loss, **(c)** Exploration and RMSE.

Figure 14 presents the additional validation under a demanding trajectory profile. In Figure 14a, all controllers track the combined half-lemniscate and closed-zigzag path, where the zigzag section introduces sharp direction changes. In Figure 14b, Alpha–Beta Minimax achieves the lowest ${\text{RMSE}}_{xy}$ of 0.15091 m, while the proposed SSS*–DDQN maintains competitive performance with ${\text{RMSE}}_{xy}=0.15605$ m and ${\text{MAE}}_{xy}=0.10606$ m. In Figure 14c, Alpha–Beta Minimax achieves the lowest ${\text{RMSE}}_{\psi }$ of 0.54181 rad, whereas QMDP-PER-DDQN yields the best reward of −5630.71 . Overall, the results confirm that the proposed SSS*–DDQN remains robust under sharp trajectory changes, although some baseline methods perform better in individual metrics.

**FIGURE 14:**
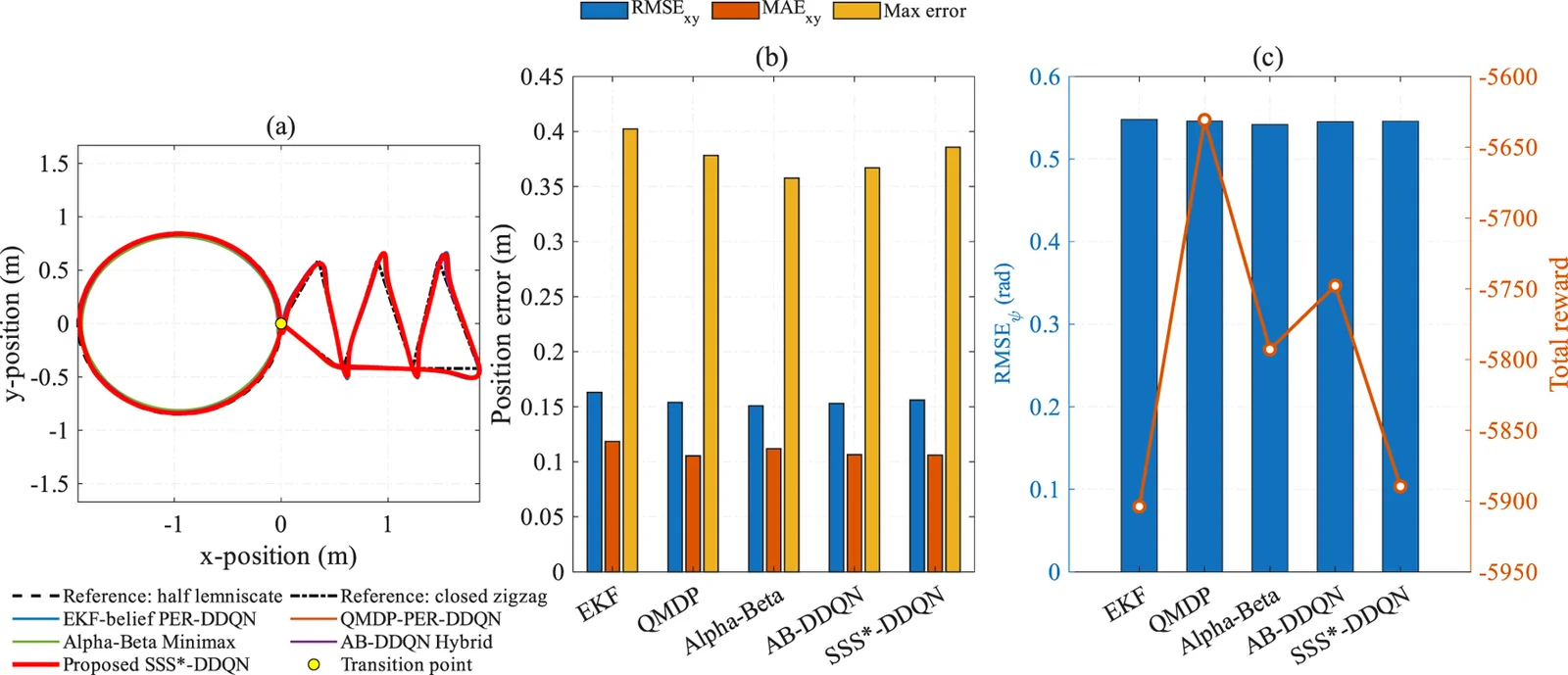
Additional validation under a demanding trajectory profile: **(a)** half-lemniscate and closed-zigzag tracking response, **(b)** position tracking metrics under sharp path changes, and **(c)** heading-error and reward response.

### 5.1 Statistical validation under stochastic noise and dropout

To further validate robustness under stochastic sensing conditions, each controller was evaluated over multiple independent simulation trials using different random seeds for measurement noise, sensor dropout, and disturbance realization. Since Table 7 reports the representative performance and final ranking, the additional statistical validation focuses only on run-to-run variation. Therefore, the mean, standard deviation, and 95% confidence interval are reported for the main tracking metrics. The sample mean and standard deviation are computed using Equations 121, 122:

$$\bar{x}=\frac{1}{N}\overset{N}{\underset{i=1}{\sum }}x_{i},$$ (121)

$$\sigma_{x}=\sqrt{\frac{1}{N-1}\overset{N}{\underset{i=1}{\sum }}{\left(x_{i}-\bar{x}\right)}^{2}},$$ (122)

where N is the number of independent trials and $x_{i}$ is the metric obtained in the i th trial. The 95% confidence interval is calculated using Equation 123:

$${CI}_{95\%}=\bar{x}\pm 1.96\frac{\sigma_{x}}{\sqrt{N}}.$$ (123)

Figure 15 presents the statistical validation of the five controllers over multiple stochastic trials. In Figure 15a, the proposed SSS*–DDQN achieves the lowest ${\text{RMSE}}_{xy}$ of 0.01847±0.00132 m, with a 95% CI of 0.01708–0.01986, followed by AB–DDQN Hybrid with 0.01879±0.00139 m. In Figure 15b, the proposed method also achieves the lowest ${\text{MAE}}_{xy}$ of 0.01554±0.00128 m, with a 95% CI of 0.01420–0.01688, followed by AB–DDQN Hybrid with 0.01622±0.00140 m. In Figure 15c, EKF-belief PER-DDQN and QMDP-PER-DDQN show higher run-to-run variation, whereas AB–DDQN Hybrid and proposed SSS*–DDQN maintain lower variation. Although Alpha–Beta Minimax has very small standard deviation, its mean ${\text{RMSE}}_{xy}$ and ${\text{MAE}}_{xy}$ are higher. Overall, the proposed SSS*–DDQN achieves the lowest mean ${\text{RMSE}}_{xy}$ and ${\text{MAE}}_{xy}$ , with low standard deviation and narrow confidence intervals. This confirms that the proposed controller maintains consistent tracking performance under stochastic noise, dropout, and disturbance conditions.

**FIGURE 15:**
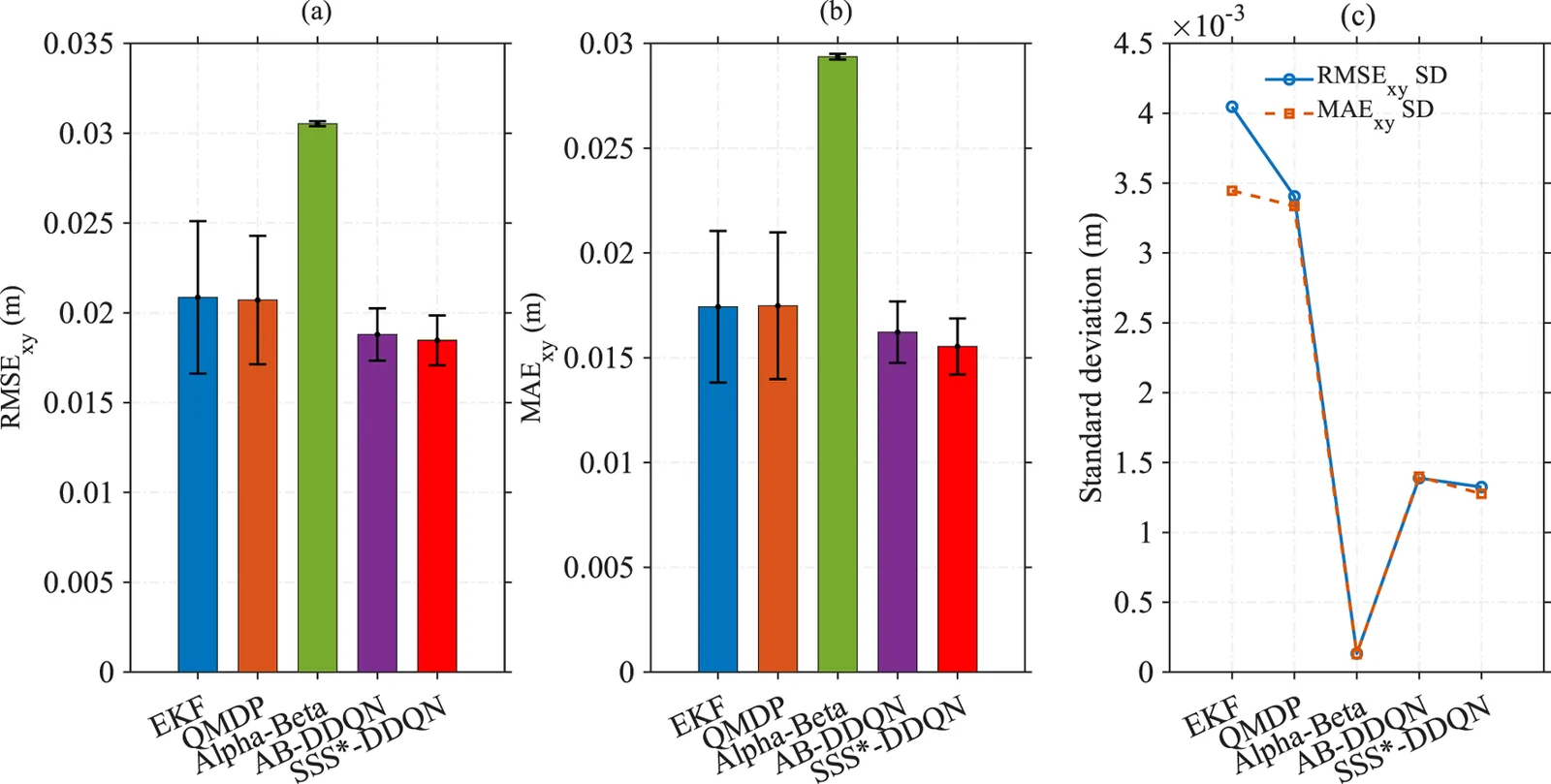
Statistical validation over multiple stochastic trials. **(a)** RMSExy mean and 95% CI, **(b)** MAExy mean and 95% CI, **(c)** Run-to-run variation.

Table 5 reports the statistical validation over multiple independent stochastic trials with different noise, dropout, and disturbance realizations. The proposed SSS*–DDQN achieves the lowest mean ${\text{RMSE}}_{xy}$ of 0.01847 m and the lowest mean ${\text{MAE}}_{xy}$ of 0.01554 m. It also maintains low standard deviation values of 0.00132 m and 0.00128 m for ${\text{RMSE}}_{xy}$ and ${\text{MAE}}_{xy}$ , respectively. The corresponding 95% confidence intervals, [0.01708–0.01986] for ${\text{RMSE}}_{xy}$ and [0.01420–0.01688] for ${\text{MAE}}_{xy}$ , confirm consistent tracking performance under stochastic sensing uncertainty.

**TABLE 5:** Statistical validation of controller performance over multiple independent stochastic trials. The best mean tracking results are highlighted in bold.

Controller	${RMSE}_{xy}$ Mean ± sd	${RMSE}_{xy}$ 95% CI	${MAE}_{xy}$ Mean ± sd	${MAE}_{xy}$ 95% CI
EKF-belief PER-DDQN	0.02086±0.00405	[0.01662, 0.02511]	0.01743±0.00345	[0.01382, 0.02105]
QMDP-PER-DDQN	0.02071±0.00341	[0.01714, 0.02429]	0.01748±0.00334	[0.01398, 0.02098]
Alpha–Beta Minimax	0.03053±0.00013	[0.03039, 0.03067]	0.02937±0.00013	[0.02923, 0.02950]
AB–DDQN Hybrid	0.01879±0.00139	[0.01734, 0.02025]	0.01622±0.00140	[0.01475, 0.01769]
Proposed SSS*–DDQN	0.01847±0.00132	[0.01708, 0.01986]	0.01554±0.00128	[0.01420, 0.01688]

### 5.2 Computational complexity, real-time suitability, and scalability

The proposed SSS*–DDQN controller has higher computational cost than EKF-belief PER-DDQN and QMDP-PER-DDQN because it performs online solution-space best-first search in addition to DDQN value evaluation. However, the complexity is controlled through top- K action ranking, shallow lookahead, bounded OPEN-list management, beta-bound pruning, delayed MIN-sibling refinement, limited belief samples, and selective secondary lookahead. As shown in Table 4, the proposed SSS*–DDQN refined $3.9494\times 10^{5}$ nodes, generated $7.9705\times 10^{5}$ nodes, pruned $2.5382\times 10^{5}$ nodes, and achieved a pruning ratio of 64.27% , with an execution index of $1.4822\times 10^{6}$ . Although this cost is higher than Alpha–Beta Minimax and AB–DDQN Hybrid, Table 7 confirms that the proposed method achieved the best overall rank, with the lowest ${\text{RMSE}}_{xy}$ of 0.01906 m and ${\text{MAE}}_{xy}$ of 0.016074 m . The computational load mainly depends on the number of top-ranked actions, search depth, disturbance branches, belief samples, and OPEN-list size. Therefore, for real-time deployment, the method can be scaled using adaptive search budget, uncertainty-triggered SSS* activation, reduced top- K actions, fewer belief samples, parallel/GPU evaluation, and fallback to QMDP-PER-DDQN or EKF-belief PER-DDQN when computational resources are limited. Figure 12 further confirms that the proposed SSS*–DDQN uses higher but more structured search effort to improve robust action selection under partial observability.

In addition to the search-node statistics reported in Table 6, quantitative single-step runtime profiling was performed to evaluate the real-time suitability of the five controllers. The controller algorithms were implemented using MATLAB, while the MATLAB–NVIDIA Isaac Sim co-simulation arrangement shown in Figure 16 was used to represent the four-Mecanum-wheel robot environment and its trajectory-tracking response through the ROS 2 interface. Three independent stochastic trials were conducted with 1,000 control steps per trial. The first 50 steps of each trial were excluded to reduce initialization and computational warm-up effects. Therefore, a total of (Equation 124)

$$N_{\text{timed}}=3\left(1000-50\right)=2850.$$ (124)

control steps were analyzed for each controller. The sampling period was $T_{s}=0.02$ s, corresponding to a nominal control frequency of 50 Hz and a single-step computational deadline of 20 ms. The measured interval covered controller-side processing from action selection to completion of the EKF-corrected control step. Table 6 summarizes the mean runtime, 95th-percentile runtime, and percentage of steps exceeding the 20 ms deadline. Figure 16 visually compares the mean and 95th-percentile execution times against the controller deadline.

**TABLE 6:** Single-step runtime profile of the five controllers. The lowest runtime and deadline-exceedance values are highlighted in bold.

Controller	Mean (ms)	P59 (ms)	Deadline exceedance (%)
EKF-belief PER-DDQN	**1.961**	**3.033**	**0.00**
QMDP-PER-DDQN	19.488	32.856	19.30
Alpha–Beta Minimax	236.430	447.980	99.79
AB–DDQN Hybrid	1,031.100	1,485.100	100.00
Proposed SSS*–DDQN	4,830.400	6,587.900	100.00

**FIGURE 16:**
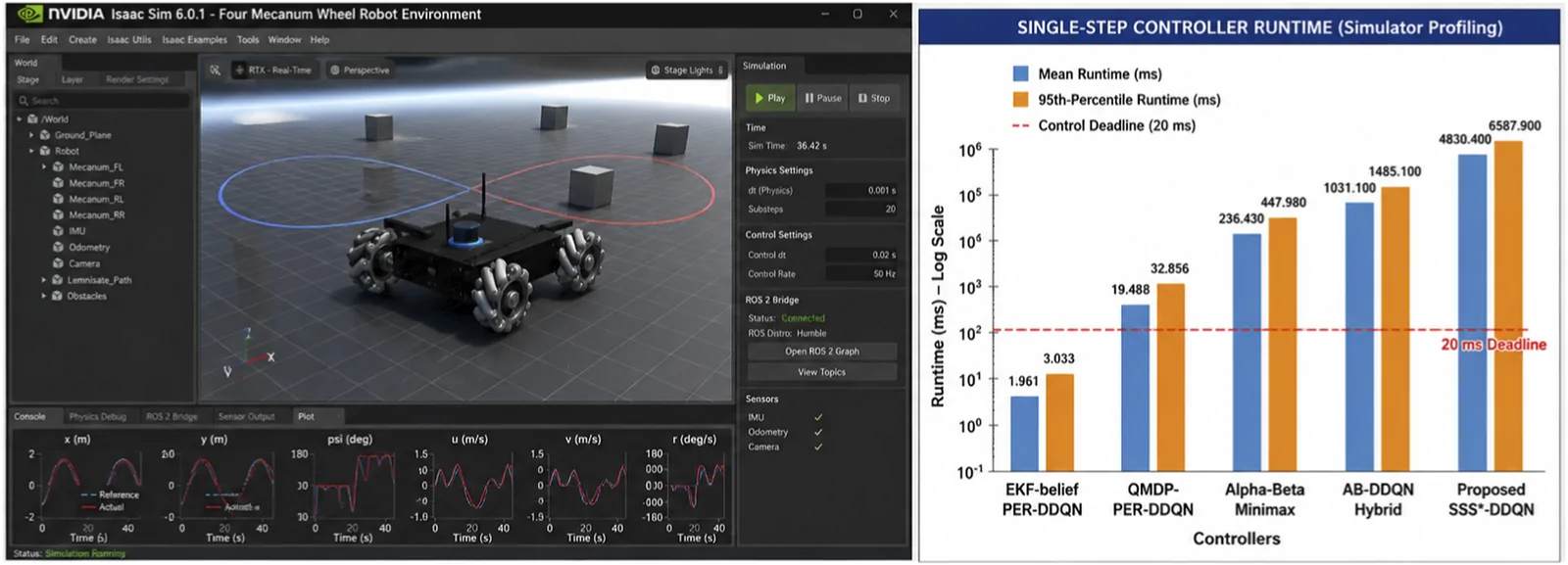
MATLAB–NVIDIA Isaac Sim co-simulation environment and single-step controller-runtime comparison. The left panel shows the four-Mecanum-wheel robot simulation and trajectory response, while the right panel compares the mean and 95th-percentile controller runtimes against the 20 ms control deadline.

The EKF-belief PER-DDQN controller achieved a mean runtime of 1.961 ms and a 95th-percentile runtime of 3.033 ms. All evaluated steps were completed within the 20 ms deadline, demonstrating its suitability for 50 Hz execution. QMDP-PER-DDQN achieved a mean runtime of 19.488 ms, which was slightly below the nominal deadline. However, its 95th-percentile runtime increased to 32.856 ms, and 19.30% of its control steps exceeded the deadline. Therefore, its average runtime does not ensure deterministic real-time execution. The search-based controllers required substantially greater computational effort. Alpha–Beta Minimax and AB–DDQN Hybrid recorded mean runtimes of 236.430 ms and 1,031.100 ms, respectively. Their high runtimes resulted from recursive disturbance-branch evaluation and repeated terminal-node processing. The proposed SSS*–DDQN required a mean runtime of 4,830.400 ms and a 95th-percentile runtime of 6,587.900 ms. All evaluated SSS*–DDQN steps exceeded the 20 ms deadline. As illustrated in Figure 16, the computational demand increased considerably from direct belief-based DDQN action selection to solution-space best-first search. Component-level profiling showed that SSS* OPEN-list refinement required an average of 4,772.2 ms and accounted for approximately 98.80% of the total SSS*–DDQN execution time. Root QMDP-DDQN action ranking and secondary lookahead contributed approximately 0.54% and 0.65%, respectively, whereas the EKF belief update contributed less than 0.01%. Thus, the dominant computational bottleneck was the repeated refinement of LIVE and SOLVED nodes in the SSS* OPEN priority queue rather than belief-state estimation. These results establish a clear trade-off between uncertainty-aware search refinement and computational feasibility. The present full-search SSS*–DDQN configuration is suitable for simulation-based validation, offline analysis, and supervisory decision-making applications, but it does not currently satisfy the 50 Hz real-time control requirement.

### 5.3 Overall controller ranking and discussion

Figure 17 presents the overall controller ranking and performance comparison using the values reported in Table 7. In Figure 17a, the ${\text{RMSE}}_{xy}$ values are 0.01906 m for the proposed SSS*–DDQN, 0.02189 m for AB–DDQN Hybrid, 0.02236 m for QMDP-PER-DDQN, 0.028422 m for EKF-belief PER-DDQN, and 0.030639 m for Alpha–Beta Minimax. In Figure 17b, the ${\text{RMSE}}_{\psi }$ values are 0.004327 rad, 0.0087679 rad, 0.016244 rad, 0.0088879 rad, and 0.0041977 rad, respectively. In Figure 17c, the ${\text{MAE}}_{xy}$ values are 0.016074 m, 0.019558 m, 0.020742 m, 0.025891 m, and 0.029516 m, respectively. In Figure 17d, the maximum tracking errors are 0.070695 m, 0.072498 m, 0.069297 m, 0.074727 m, and 0.074918 m, respectively. In Figure 17e, the mean belief uncertainty, measured by tr(P), is 0.002718 for all controllers. In Figure 17f, the final scores are 0.55376 for the proposed SSS*–DDQN, 0.68303 for AB–DDQN Hybrid, 0.71175 for QMDP-PER-DDQN, 0.80045 for EKF-belief PER-DDQN, and 0.88876 for Alpha–Beta Minimax, corresponding to ranks 1 to 5, respectively.

**FIGURE 17:**
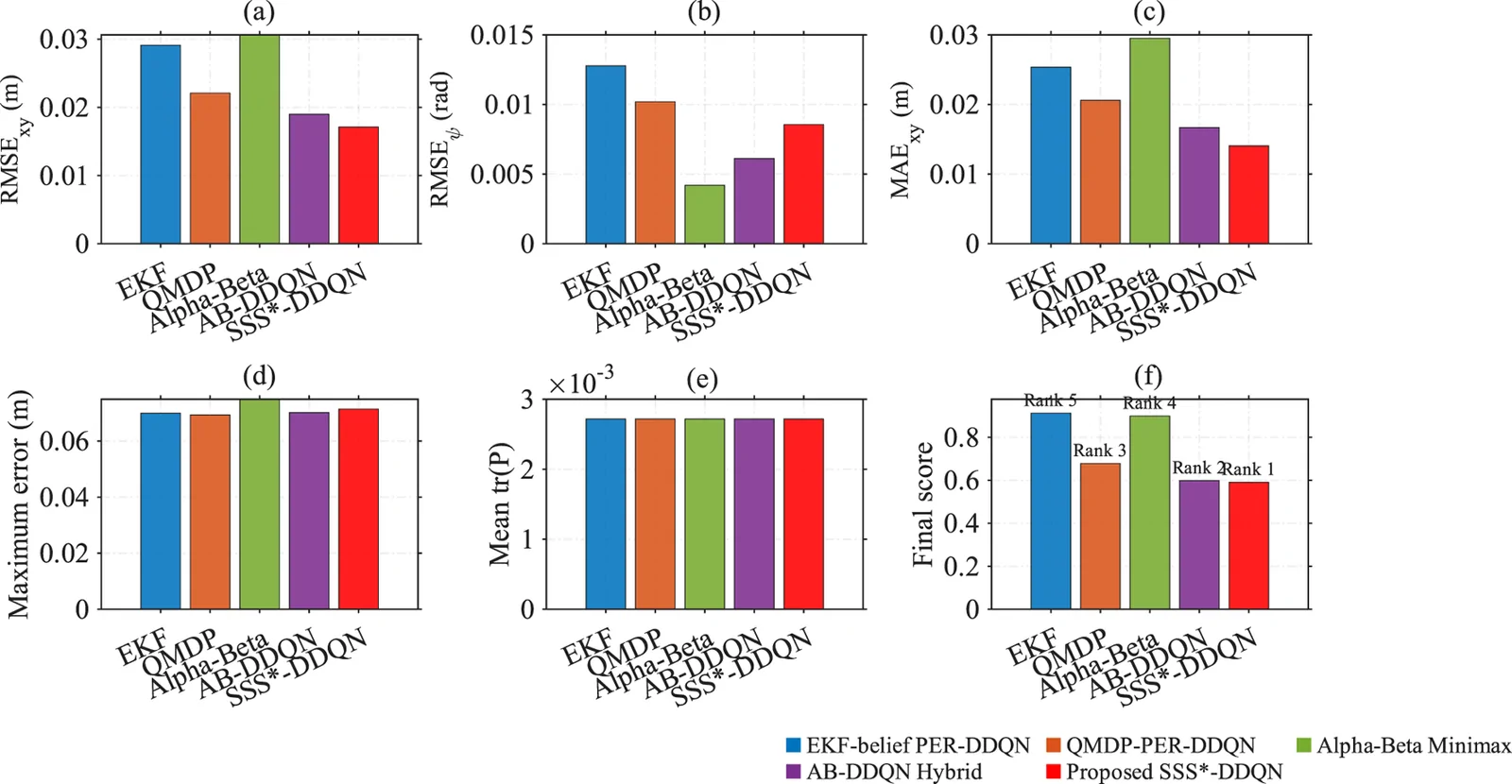
Overall controller ranking and performance comparison. **(a)** RMSE position error, **(b)** RMSE heading error, **(c)** Mean absolute error, **(d)** Maximum tracking error, **(e)** Mean belief uncertainty, **(f)** Final ranking score.

**TABLE 7:** Overall controller ranking based on tracking error, belief trace, reward, and final score. The best result in each performance metric is highlighted in bold.

Controller	${RMSE}_{xy}$ **(m)**	${RMSE}_{\psi }$ **(rad)**	${MAE}_{xy}$ **(m)**	**Max. Error** **(m)**	**Mean** **tr** (P)	**Reward**	**Final score**	**Rank**
Proposed SSS*–DDQN	0.01906	**0.004327**	**0.016074**	**0.070695**	**0.002718**	**1843.6**	**0.55376**	**1**
AB–DDQN Hybrid	**0.02189**	**0.0087679**	**0.019558**	**0.072498**	**0.002718**	**1798.8**	**0.68303**	**2**
QMDP-PER-DDQN	**0.02236**	**0.016244**	**0.020742**	**0.069297**	**0.002718**	**1852.9**	**0.71175**	**3**
EKF-belief PER-DDQN	**0.028422**	**0.0088879**	**0.025891**	**0.074727**	**0.002718**	**1780.3**	**0.80045**	**4**
Alpha–Beta Minimax	**0.030639**	**0.0041977**	**0.029516**	**0.074918**	**0.002718**	**1,675.6**	**0.88876**	**5**

Overall, the proposed SSS*–DDQN achieves the best final rank because it provides the most balanced performance across position tracking accuracy, heading accuracy, mean absolute error, maximum error, and reward. Although QMDP-PER-DDQN achieves the highest reward and Alpha–Beta Minimax achieves the lowest ${\text{RMSE}}_{\psi }$ , their weaker performance in the other metrics reduces their overall ranking. Therefore, the proposed SSS*–DDQN offers the most effective overall controller performance under partial observability.

### 5.4 Limitations and practical deployment considerations

Although the proposed SSS*–DDQN framework improves robust action selection under partial observability, it may underperform in situations where computational resources are severely limited. Since the method performs online best-first solution-space refinement, it requires more computation and memory than direct EKF-belief PER-DDQN or QMDP-PER-DDQN action selection. Therefore, under strict real-time deadlines, low-power embedded processors, limited memory, very high control frequencies, large belief-sample sets, or large disturbance-branch sets, simpler controllers may be more suitable.

The proposed method also provides limited additional benefit in simple low-uncertainty tracking tasks where direct DDQN-based or QMDP-based action selection is already sufficient. Its advantage is expected to be more significant when the robot operates under noisy measurements, sensor dropout, external disturbances, and short-horizon decision uncertainty. For real-world deployment, adaptive search budgeting, uncertainty-triggered activation, reduced search depth, limited belief sampling, parallel implementation, and fallback control are recommended to balance tracking robustness and computational efficiency.

### 5.5 Ablation study of proposed framework components

Figure 18 and Table 8 present the component-wise ablation analysis of the proposed SSS*–DDQN framework. In Figure 18a, the module-availability matrix shows that the proposed SSS*–DDQN is the only controller that combines all six components: EKF estimation, QMDP belief sampling, DDQN learning, SSS* refinement, beta-bound pruning, and secondary lookahead. In Figure 18b, the proposed SSS*–DDQN achieves the lowest RMSE_*xy*_ of 0.01906 m and MAE_*xy*_ of 0.016074 m, confirming improved tracking accuracy. In Figure 18c, the proposed method also achieves the strongest search-refinement contribution, with 64.27% pruning and 36,383 secondary-lookahead operations. Overall, the ablation results confirm that the improvement is not due to a single module alone. EKF provides stable belief estimation, QMDP improves uncertainty-aware action evaluation, DDQN supplies learned value guidance, SSS* refines promising action clusters, beta-bound pruning reduces unnecessary expansion, and secondary lookahead improves final action robustness.

**FIGURE 18:**
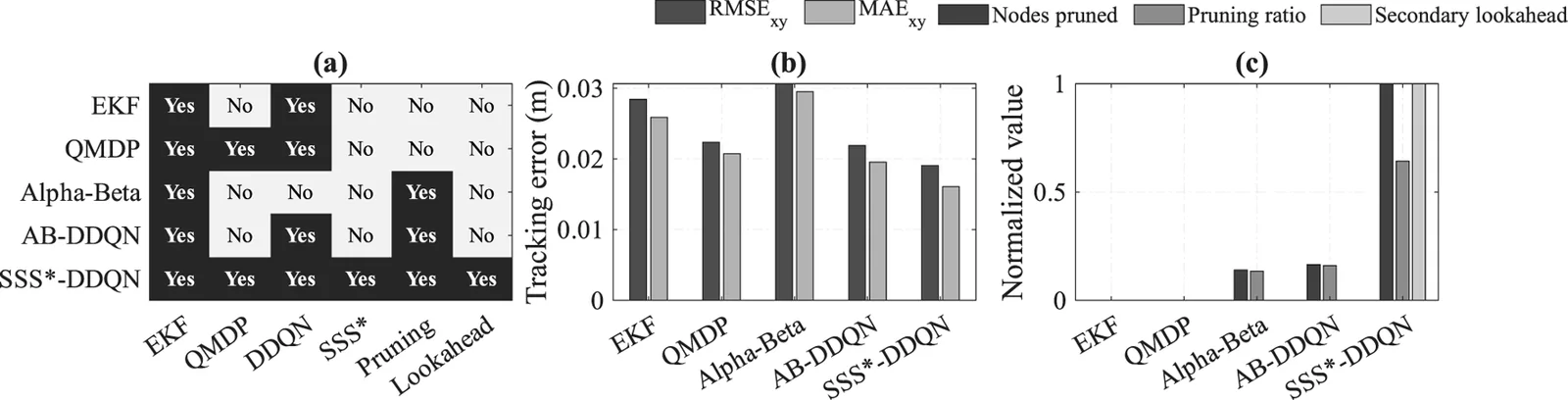
Component-wise ablation analysis of the proposed SSS*–DDQN framework. **(a)** Component availability matrix **(b)** Tracking accuracy contribution **(c)** Search refinement contribution.

**TABLE 8:** Component-wise ablation study of the proposed SSS*–DDQN framework.

Controller variant	EKF	QMDP	DDQN	SSS*	Pruning	Lookahead	Observed contribution
EKF-belief PER-DDQN	Yes	No	Yes	No	No	No	Belief-state filtering with DDQN action selection
QMDP-PER-DDQN	Yes	Yes	Yes	No	No	No	Adds belief sampling for robust action evaluation
Alpha–Beta Minimax	Yes	No	No	No	Yes	No	Provides adversarial search and strong heading accuracy
AB–DDQN Hybrid	Yes	No	Yes	No	Yes	No	Combines Alpha–Beta search with DDQN-based leaf evaluation
Proposed SSS*–DDQN	Yes	Yes	Yes	Yes	Yes	Yes	Combines EKF estimation, QMDP belief sampling, DDQN value guidance, SSS* refinement, pruning, and secondary lookahead

## 6 Conclusion

In this study, we presented an SSS*–DDQN solution-space best-first search controller for robust trajectory tracking of a four-Mecanum-wheel mobile robot under partial observability. The proposed method combines EKF belief estimation, QMDP belief sampling, DDQN value evaluation, and SSS* OPEN-list refinement with LIVE/SOLVED node handling. The results show that the proposed controller achieved the best overall rank with ${\text{RMSE}}_{xy}=0.01906\;m$ , ${\text{MAE}}_{xy}=0.016074\;m$ , and cumulative reward of 1843.6. Statistical validation further confirmed consistent performance under stochastic noise and dropout.

The improvement is mainly due to belief-aware filtering, learned value estimation, and best-first corrective-action refinement. However, the method requires higher computation than simpler DDQN-based controllers. Therefore, practical deployment should use adaptive search budgeting, reduced belief samples, parallel implementation, and fallback control under limited resources. Future work will focus on hardware testing, real-time profiling, ablation studies, additional trajectory cases, and comparison with PPO, SAC, TD3, MPC, and MCTS-based methods.

## Data Availability

The original contributions presented in the study are included in the article/supplementary material, further inquiries can be directed to the corresponding authors.
